# Conserved DNA Methylation Signatures in Early Maternal Separation and in Twins Discordant for CO_2_ Sensitivity

**DOI:** 10.1038/s41598-018-20457-3

**Published:** 2018-02-02

**Authors:** Francesca Giannese, Alessandra Luchetti, Giulia Barbiera, Valentina Lampis, Claudio Zanettini, Gun Peggy Knudsen, Simona Scaini, Dejan Lazarevic, Davide Cittaro, Francesca R. D’Amato, Marco Battaglia

**Affiliations:** 1 Centre for Translational Genomics and Bioinformatics, Istituto di Ricovero e Cura a Carattere Scientifico (IRCCS), San Raffaele Scientific Institute, Milan, Italy; 2 Institute of Cell Biology and Neurobiology, National Research Council, Rome, Italy; 3 Department of Oncology, University of Milan, Milan, Italy; 4 The Norwegian Institute of Public Health Department of Genetics, Environment and Mental Health, Oslo, Norway; 5 Department of Psychology, Sigmund Freud University, Milan, Italy; 6 Department of Psychiatry, the University of Toronto, Toronto, Canada; 7 Division of Child, Youth and Emerging Adulthood, Centre for Addiction and Mental Health, Toronto, Canada; 8 Present Address: National Institute on Drug Abuse, Medication Development Program Molecular Targets and Medications Discovery Branch, Intramural Research Program, NIH, Baltimore, USA

## Abstract

Respiratory and emotional responses to blood-acidifying inhalation of CO_2_ are markers of some human anxiety disorders, and can be enhanced by repeatedly cross-fostering (RCF) mouse pups from their biological mother to unrelated lactating females. Yet, these dynamics remain poorly understood. We show RCF-associated intergenerational transmission of CO_2_ sensitivity in normally-reared mice descending from RCF-exposed females, and describe the accompanying alterations in brain DNA methylation patterns. These epigenetic signatures were compared to DNA methylation profiles of monozygotic twins discordant for emotional reactivity to a CO_2_ challenge. Altered methylation was consistently associated with repeated elements and transcriptional regulatory regions among RCF-exposed animals, their normally-reared offspring, and humans with CO_2_ hypersensitivity. In both species, regions bearing differential methylation were associated with neurodevelopment, circulation, and response to pH acidification processes, and notably included the *ASIC2* gene. Our data show that CO_2_ hypersensitivity is associated with specific methylation clusters and genes that subserve chemoreception and anxiety. The methylation status of genes implicated in acid-sensing functions can inform etiological and therapeutic research in this field.

## Introduction

For almost every living species, heightened CO_2_ concentration ([CO_2_]) in inhaled air is a potent adversative stimulus. As a result, the physiological and behavioural responses to increased [CO_2_] map one of the most basic and universal alarm/avoidance systems within the realm of biology^1^. Once it permeates the blood-brain barrier, CO_2_ is hydrolysed and dissociated into HCO_3_^−^ and H^+^, causing the temporary acidification of brain extracellular fluids^2^. In all mammals, heightened [CO_2_] evokes a compensatory hyperventilation to restore physiological pH, increased arousal/fear, and is typically followed by active avoidance^2,3^.

Sensitivity to CO_2_ is particularly enhanced in humans with panic disorder (PD), amongst whom exaggerated respiratory and emotional reactivity to blood-acidifying CO_2_-enriched air mixtures^4,5^, increased propensity to asthma and finely altered ventilator physiology^6^, and sudden increase of CO_2_ partial pressure preceding spontaneous panic attacks^7^ are well documented. Together with augmented brainstem activation to hypercarbia^8^ and evidence of increased brain lactate concentrations^9,10^, these data are compatible with central hypersensitivity to acidification^9^ or abnormal brain pH regulation in PD, corroborate a theory of CO_2_ hypersensitivity^2^ and the validity of a more severe and prevalent respiratory subtype of PD^11^.

Inasmuch as there is between-individuals’ variation for the degree of hyperventilation and arousal/fear induced by CO_2_ inhalation^2,3^, the hyperventilation and/or panicky responses to CO_2_ proper of people at risk for PD are best conceptualised as extreme variants of a physiologic, quantitative phenotype that can be comprehensively identified as the CO_2_ hypersensitivity trait^1,12,13^.

Human twins data^14,15^ show that panicky reactions to a CO_2_ inhalation challenge, PD, and the developmental antecedent of childhood separation anxiety^16^ share a largely overlapping genetic background. Childhood parental loss (including separation from parents, divorce, death) adds significantly to this covariation^14^, and early-life adversities heighten individual responsiveness to CO_2_ via gene-by-environment interaction mechanism (GXE)^17^.

Independent groups transferred this knowledge to the animal laboratory, and responses to CO_2_ and brain acidification became translational tools to investigate PD by proxies in animals^18–22^. Neonatal maternal separation in rats^19^, or the repeated cross fostering (RCF^20^) of mouse pups to lactating females other than the biological mother, stably alter offspring’s CO_2_ sensitivity, as shown by larger (40–50% increase^19,20,23^) tidal volume and larger minute volume responses to CO_2_ challenges, compared to normally-reared control (CT) animals^19,20,24^. Similar to human data^4^, RCF-induced CO_2_ hypersensitivity is not associated with HPA axis dysregulation^20,24^. Also, RCF does not affect sociability/social preference, exploratory anxiety^24^ or normal air breathing^20,24^.

A major impetus to the present study was provided by evidence that, similarly to human CO_2_ hypersensitivity^17^, RCF-induced CO_2_ hypersensitivity is sensitive to GxE mechanisms^20^, as indicated by the enhanced heritability of tidal volume increment during 6%CO_2_ breathing among RCF animals^20^. Moreover, recent histone marks’ and transcriptome profiling of the medulla oblongata (MO, a pivotal brainstem site of chemoreception^2^) of RCF vs. control animals showed the enrichment of genes subserving chemoreception, learning and fear, and neurodevelopment^25^. Specifically, altered chromatin structure and enhanced expression of the Acid Sensing Ion Channels1 (ASIC1^25^ among RCF animals^25^ were consistent with current models of CO_2_ sensitivity and PD^3,21,26^, and with the finding of altered nociception associated with the RCF procedure^25^.

No study has concurrently investigated the DNA methylation patterns of humans who overreact to a CO_2_ challenge, and animals that develop CO_2_ hypersensitivity following early maternal separation. Such inter-species comparisons of the epigenomes become viable by novel computational clustering approaches. These allow for direct comparisons of differentially-methylated regions in humans and mice while taking the species’-specific functional context into consideration (see, *e.g*.^27^).

In the present study, we investigated the DNA methylation profiles of animals exposed to the RCF paradigm, and of human monozygotic twin pairs discordant for CO_2_ hypersensitivity (MZD), together with the patterns of inheritance of such regions, and the RCF-associated intergenerational transmission of CO_2_ hypersensitivity. Our principal aim was the identification of conserved regulatory regions associated with CO_2_ hypersensitivity in the two species.

Our study found altered DNA methylation signals in genomic regions that subserve neurodevelopment and chemoreception conserved among CO_2_-hypersensitive twins and RCF-exposed mice.

## Results

### Phenotypes

**Figure 1:**
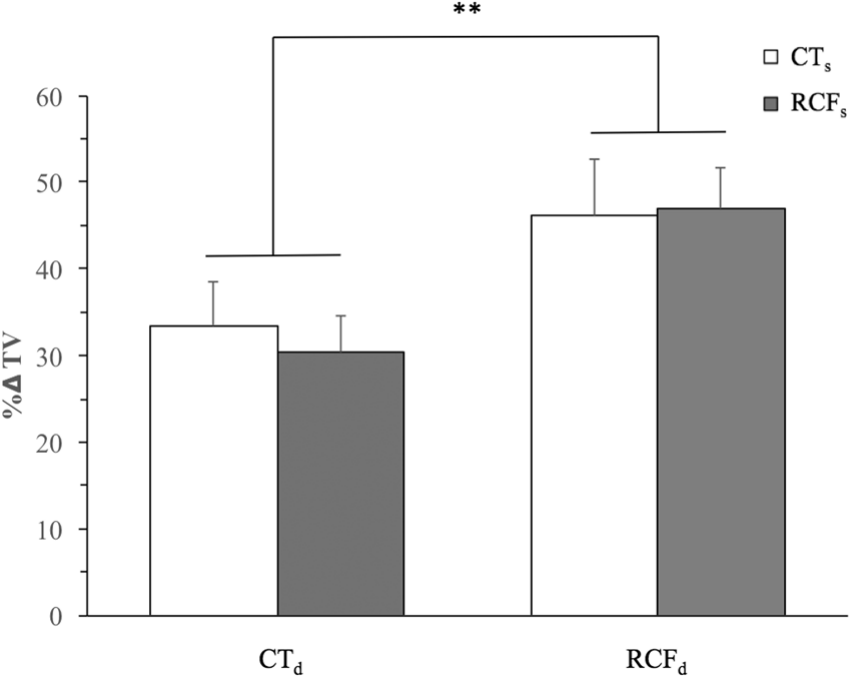
Intergenerational transmission of CO_2_ hypersensitivity among normally-reared F1 pups.The figure shows the role of F0-RCF vs. F0-CT maternal lineage, as specified in abscissa: a grey histogram indicates crossing of a RCF/CT dam with an RCF F0-sire, and a white histogram indicates crossing of a RCF/CT dam with a CT F0-sire. At post-natal day (PND) 16–22, F1 pups (n = 32) resulting from all the 4 possible mating combinations of F0-RCF or F0-CT dams and sires (8 pups/parental mating combination, balanced by sex, see also *Methods* section) were assessed for their respiratory physiology during normal air and CO_2_-enriched air breathing. A two-way ANOVA of Δ%TV responses to 6%CO_2_-enriched air mixture vs. normal air showed a significant effect of F0 maternal treatment (F_1,28_ = 8.65, p = 0.007), but no significant effect of F0 paternal treatment (p = 0.75), or interaction of F0 maternal-by-paternal treatment (p = 0.80). Females’ responses (Δ%TV: 37.07 ± 3.56) did not differ from males’ responses (Δ%TV: 41.69 ± 4.28, t = −0.82, DF = 30, p = NS).

A significantly heightened increment of tidal volume (ΔTV %) in response to 6%CO_2_-enriched air was found amongst normally-reared F1 mouse pups (postnatal day: PND 16–22) (Fig. 1).

This was associated with maternal, not paternal, history of RCF. To investigate this intergenerational transmission of CO_2_ hypersensitivity from the RCF-exposed F0 parental generation to the normally-reared F1 offspring generation, we analysed the respiratory responses of F1 pups (n = 32) resulting from all the 4 possible mating combinations of F0-RCF or F0-CT dams and sires (8 pups/parental mating combination, balanced by sex, see also *Methods* section), during normal air and 6%CO_2_-enriched air breathing. A two-way ANOVA of Δ%TV responses to 6%CO_2_-enriched air mixture vs. normal air showed a significant effect of F0 maternal treatment (F_1,28_ = 8.65, p = 0.007), but no significant effect of F0 paternal treatment (p = 0.75), or interaction of F0 maternal-by-paternal treatment (p = 0.80). F1 females’ responses (Δ%TV: 37.07 ± 3.56) did not differ from males’ responses (Δ%TV: 41.69 ± 4.28, t = −0.82, DF = 30, p = NS).

This effect could not be attributed to apparent differences in maternal care, as maternal nursing and licking/grooming (Supplementary Figure S1) did not differ between F0-RCF_d_ (dams that had experienced RCF as pups), and F0-CT_d_ (dams that had been raised in standard conditions).

The presence of CO_2_ hypersensitivity among normally-reared F1 offspring of F0-RCF_d_ was consistently replicated across 3 other, independent intergenerational samples (Supplementary Table S1; Supplementary Figure S2), and was recognisable also in adulthood, as shown by comparison of respiratory responses of naïve F0 RCF and F0 CT animals at PND 75–90 (Supplementary Figure S3).

### Differential DNA methylation in intergenerational CO_2_ hypersensitivity

Whole-genome DNA methylation profiling was performed on adult female mice’s brain stems deriving from 2 experimental sets different from those employed to assess phenotypes. These were: one F0 generation (encompassing both CT and RCF females) and one F1 generation (encompassing normally-reared female offspring of: F0 RCF dams, and F0 CT dams). Differential methylation analysis yielded 976 differentially methylated regions for F0 and 759 for F1 generation (*p-*value < 0.001) (Supplementary Table S2).

### DMR analysis

A consistent pattern of overrepresentation of DMRs in regulatory regions and repeated elements emerged across the two experiments (Supplementary Figure S4a,b,c).

DMRs were found strongly associated with chromatin states related to gene expression regulation, while no prominent association with active promoters emerged (Supplementary Figure S4a**)**. Specifically, consistent DMRs enrichment was observed in association with poised enhancers for both F0 and F1. DMR enrichment in association with brain-specific superenhancers was observed in the F1 generation. Significant enrichment of DMRs in poised promoters, strong enhancers, repressed and transcribed regions was observed for the F0 experiment. (Supplementary Figure S4a). Consistently, in the F0 generation DMRs were enriched in exons and introns (p = 0.02) and were non-significantly depleted in TSS-flanking regions (p = 0.07) (Supplementary Figure S4b).

As for repeated elements, F0 and F1 DMRs were enriched in long terminal repeats (LTR) and satellite regions, and depleted in long interspersed nuclear elements (LINE) and low complexity regions (Supplementary Figure S4c). Significant enrichment in simple repeats and short interspersed nuclear elements (SINE) regions was observed for the F0 generation (Supplementary Figure S4c).

### DNA methylation clusters analysis

Methylation cluster analysis yielded 779 and 575 clusters containing at least 1 significant (p < 0.001) DMR (ccDMR) for F0 and F1 experiment, respectively (Supplementary Table S3). Median cluster size was 91708 bp for F0 and 87185 bp for F1.

Analysis of Gene Ontology enrichment showed that associated genes were related to morphogenesis, development, neurodevelopment and synaptic activity. Specifically, among top Biological Process GO terms associated with F0 experiment we found “dendrite development”, “synapse assembly”, “directional guidance of interneurons involved in migration from the subpallium to the cortex”, “noradrenergic neuron differentiation”, “neuroepithelial cell differentiation” terms (Supplementary Figure S5a; Supplementary Table S4).

Among top GOBP terms enriched in F1, we found similar processes, related to nervous system development (“forebrain development”, “central nervous system neuron axonogenesys”, “regulation of neuron projection development”) as well as electric signal transmission and circulatory system related processes (“vasoconstriction”, “cell communication by electrical coupling involved in cardiac conduction”, “blood circulation”) (Supplementary Figure S5b).

**Figure 2:**
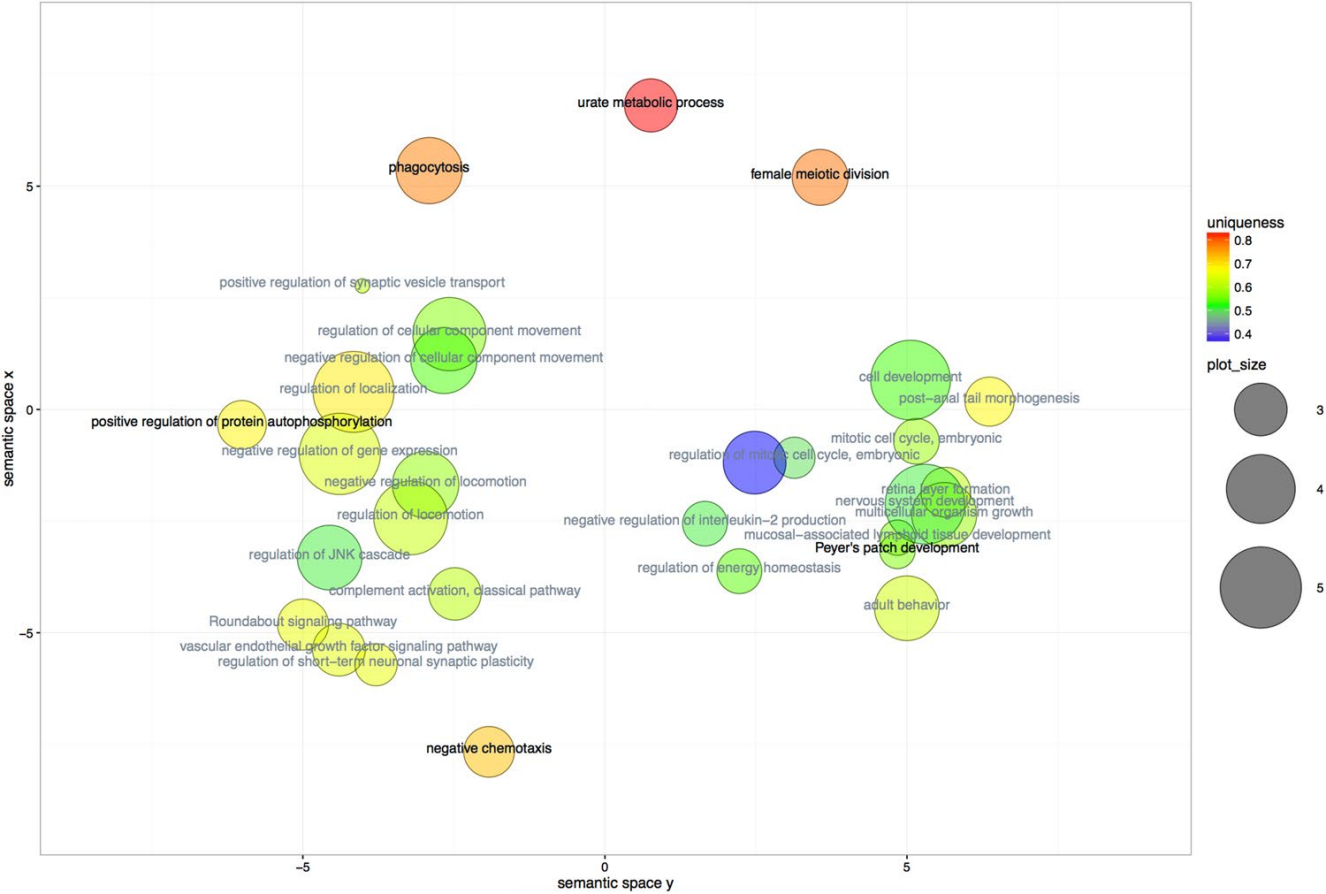
Semantic Similarity for GO terms associated with genes in intergenerationally-conserved methylation clusters (F0-F1 experiments, brain stem methylation data). Each circle symbolizes a GO term; circle size is proportional to term frequency (greater size indicates a more general term). Circle colour indicates term uniqueness (divergence from other terms) and label colour indicates dispensability (black: dispensability <0.15). Term relevance was computed by GO list ranking. Scatterplot elaborated using ReviGO (revigo.irb.hr).

There were 69 ccDMRs shared between F0 and F1, associated with 579 genes. Semantic similarity analyses highlighted several Biological Processes terms related to development, signalling pathways and ‘behavioural response’ (Supplementary Table S5; Fig. 2) suggesting that altered regulation of these pathways may underpin the manifestation and transmission of CO_2_ hypersensitivity.

Of notice, genes associated to differentially methylated clusters included the acid sensing ion channel subunit 2 (*Asic2*) gene, relevant to both acidification detection and anxiety, and Neuregulin 3 (*Nrg3*) gene, associated with “neurogenesis”, “cell motility” terms. Serotonin receptor *Htr1a* was found to be associated to “vasoconstriction” and “circulatory system process” in F1 generation’s experiment.

### Differential DNA methylation associated with CO_2_ hypersensitivity in humans

#### DMR analysis

There were 679532 methylation peaks for the MZD twins; within-pair comparisons (responder versus non-responder twin) of MZD yielded 654 DMRs (*p-*value < 10^−3^); their log-transformed fold change values (logFC) were contrasted with those of the MZ twin pairs concordant for absence of CO_2_ hypersensitivity, and yielded 223 regions with significantly higher absolute logFC values (Wilcoxon *p*-value < 0.05): 43 regions (19%) were hypomethlyated, and 190 (81%) were hypermethylated in responder twins (Supplementary Table S6).

Most DMRs’ enrichments fell in intronic regions and in short interspersed elements (SINE) (Supplementary Figure S6a,b), without significant associations with CpG islands. Further investigations of DMRs by associations with blood chromatin state signatures revealed, in agreement with the aforementioned results in mice, significant associations of DMRs with enhancer- and transcriptional activation/repression functions, rather than promotorial regions. (Supplementary Figure S6c).

#### DNA methylation clusters analysis

Clustering of twin pairs’ methylation peaks data revealed 53930 genomic regions exposed to methylation; after filtering for the presence of DMRs with p < 0.001, these were restricted to 224 ccDMRs encompassing 2244 unique genes. Functional analyses of these genes revealed associations with: nervous system development, axon guidance, blood circulation, and the Roundabout signalling pathway (Supplementary Figure S7; Supplementary Table S7). In consideration of CO_2_ hypersensitivity for which twins were investigated, the identification of terms “olfactory bulb development” (associated with *SLIT2*, *ROBO2* gene), “brain development” and “circulatory system process” (encompassing *ASIC2* and Cholinergic Receptor Muscarinic 1 *CHRM1* genes) is of particular note.

### Conserved DNA methylation features associated with CO_2_ hypersensitivity in human and mouse

**Figure 3:**
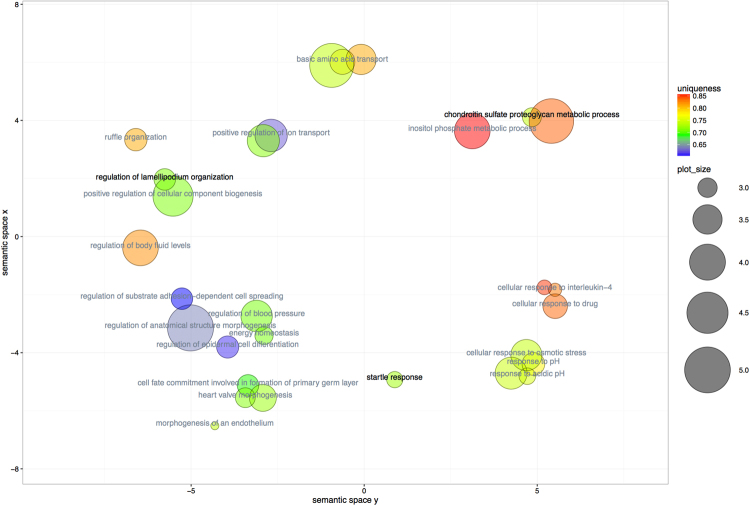
Semantic Similarity for GO terms associated with genes in methylation clusters common to MZ twins with CO_2_ hypersensitivity (blood methylation data) and RCF-exposed animals (F0 experiment, brain stem methylation data). Each circle symbolizes a GO term; circle size is proportional to term frequency (greater size indicates a more general term). Circle colour indicates term uniqueness (divergence from other terms) and label colour indicates dispensability (black: dispensability <0.15). Term relevance was computed by GO list ranking. Scatterplot elaborated by ReviGO (revigo.irb.hr).

Next we translated clusters identified in human samples to mouse genome; this allowed us to identify 37 conserved regions between human and mouse (*p-*value = 4,5*10^−4^) corresponding to 35 human ccDMRs: these regions were annotated to 828 unique genes. The top Biological Process GO terms for those genes support relevance of terms related to acidification response and circulatory system (“response to acidic pH”, “regulation of blood pressure”), associated among others to the *Asic2* gene (Fig. 3, Supplementary Table S8).

### Associations with tissue-specific chromatin state signatures and conserved clusters for CO_2_ hypersensitivity

**Figure 4:**
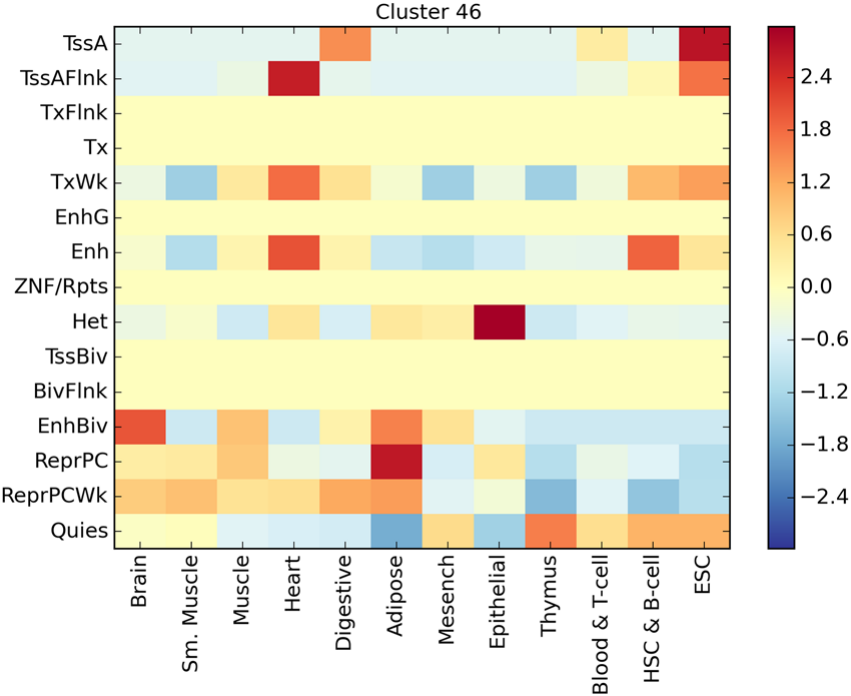
Heatmap representing relative chromatin state frequency for Cluster 46 (ASIC2-associated) Enrichment of tissue specific chromatin states in ASIC2-associated ccDMR. Color represent z-score of enrichment (red) or depletion (blue) of overlap with tissue-specific chromatin states. The ccDMR is positively associated with gene activation in ESC and enhancer function in Brain.

In order to assess tissue specificity and functional relevance of the epigenetic imprinting on the 35 human conserved ccDMRs, we annotated them to known epigenetic footprints using ENCODE annotations^28^. To this end, we calculated an enrichment score for 15 chromatin states in 12 main tissues (see Supplementary Methods) and assigned each cluster to the tissue in which they are mostly overlapping activation marks (Supplementary Table S9). We then grouped by similarity such annotations, yielding 4 main groups of ccDMRs (Supplementary Figure S8; Supplementary Table S9), characterized by their most frequently activated tissue (Supplementary Table S9). The ccDMRs Group 1 included cluster 46, associated to Embryonic Stem Cell tissue (ESC), and encompassing the *ASIC2* gene. (Fig. 4 and Supplementary Figure S9).

## Discussion

Early interference with maternal cares via the RCF paradigm is associated with intergenerational transmission of CO_2_ hypersensitivity from the RCF-exposed F0 generation, to the normally-reared F1 progeny via the maternal line. In keeping with previous research with the RCF paradigm^20,24^, this could not be ascribed to differential maternal cares provided by F0-RCF females, and together with the longitudinal stability of CO_2_ hypersensitivity among naïve F1-RCF adults, it supports a role for DNA methylation in establishing the persistence of a ‘biological memory’ for CO_2_ hypersensitivity. Evidence of GxE effects in human^17^ and RCF-induced^20^ CO_2_ hypersensitivity had already been provided by biometric-quantitative approaches, with early-life adversities playing a pivotal role^12^. The present study with the RCF paradigm is now showing that DNA methylation is one plausible molecular mechanism to explain such GxE effects.

Most studies of epigenetic changes associated with early-life interferences/adversities and the ensuing anxious phenotypes followed candidate gene approaches, often focusing on the HPA axis^29^. Here, based on the notions that altered methylation occurs in a coordinated manner over large genomic regions^30–32^ and that DNA methylation patterns^33^ and regulatory networks^34^ are conserved in mammals, we adopted a whole-genome clustering approach to uncover human and murine similarities relevant to the interspecies trait of CO_2_ sensitivity. This yielded conserved methylation clusters spanning over significant DMRs (ccDMR) in regulatory regions, for both animal (F0 and F1 experiments) and human (MZD twins) data. Our findings of inter-species’ and inter-tissues’ overlap of ccDMRs’ are both noteworthy and potentially critical, as methylation is tissue-specific^28,35–37^ and blood is a heterogeneous tissue^38^: recent data however, support blood as a valid proxy for brain methylation states^39–42^. The functional annotation of genes associated within the conserved clusters pointed to neurodevelopment, morphogenesis/differentiation and -most relevant to CO_2_ hypersensitivity- chemoreception/blood pressure regulation. Direct inference of gene regulation by DNA methylation in remote regulatory regions is elusive to address^43,44^ and a direct, mechanistic exploration of our findings in relation to CO_2_ hypersensitivity is beyond the aims of this study. Nevertheless, some genes -such as *ASIC2*, associated with our ccDMRs- are immediately relevant for CO_2_ hypersensitivity and altered nociception, that are both RCF-associated phenotypes^20,25^. ASICs are activated by H^+^: they are crucial for respiratory chemoreception as they are primarily involved in the detection of pH changes in extracellular fluids^45,46^, including hypercarbia-driven CNS acidification^47,48^. The ASIC1 and ASIC2 dimers drive pH detection^46^, with ASIC subunits’ interplays and H^+^-gated currents bridging anxiety physiology to pathophysiology^49^. Coherently, loss of ASIC2/ASIC1 reduce unconditioned (‘freezing’ responses to CO_2_), and conditioned (contextual fear) anxious responses^50,51^. Moreover, the *ASIC1* gene was found associated with human PD^52^ in a genome-wide association study, and *ASIC1* and *ASIC2* polymorphisms are associated with anxious responses to CO_2_ challenges^53^, PD^52,54^, amygdala volume and activity^54^. Our findings with *ASIC2* are thus consonant with the notion that brainstem’s responses to acidification are subserved by multiple systems^55^, and non-mutually exclusive mechanisms of RCF-induced CO_2_ hypersensitivity.

Additional, conserved ccDMRs associated with intergenerational transmission of CO_2_ hypersensitivity were in genomic regions related to neuronal morphogenesis/development, and calcium channel activity. The ccDMR associated with *Nrg3* gene in both F0 and F1 is an example in this direction: *Nrg3*’s functions in neuroblast proliferation, neural cell migration and differentiation^56,57^ suggest that RCF can affect CNS development, and indicate these phenotypes/functions as future targets for RCF-related research.

The ccDMR associated with cholinergic receptor muscarinic1 (CHRM1) gene in the MZD experiments is relevant to both CO_2_ hypersensitivity and human PD. The ventral MO chemosensitive area is rich in muscarinic receptors, and its stimulation by CO_2_/H+ elicits hyperventilation, promptly decreased by topical application of muscarinic antagonists^58,59^. Consistently, single-dose administration of centrally active –but not of peripherally active- muscarinic antagonists blocks the response to CO_2_ commonly observed in humans with PD^60^.

A general implication of our findings is that methylation profiles can guide the early identification of subjects at risk for separation anxiety/PD, and inform prevention strategies.

Four main limitations apply. First, although our findings support the methylation of key genes associated with acid sensing and anxiety to explain matrilineal transmission of CO_2_ sensitivity in mice, they are not conclusive as to the precise epigenetic mechanism of transmission. Multigenerational replications^61^ of both the CO_2_ hypersensitive phenotype and methylation data, and germline-dependent designs^62^ will be needed to firmly validate this hypothesis. Nonetheless, matrilineal intergenerational (and transgenerational) transmission of functional responses through DNA methylation^62^ is now recognised as a key mechanism to transmit the ‘biological memory’ of adversities to the following generations before conception. This is commonly interpreted as an evolutionary response to ‘tune’ offspring against challenging environments^61^. Second, although participants originated from the largest available study of human CO_2_ sensitivity^14^, our pool of MZD pairs made a comparatively small sample, and replications in independent cohorts are needed. Also, human DNA methylation was assessed in whole blood without correcting for cellular composition, as no reference epigenome for blood-cell populations obtained by meDIP-seq is yet available. Although it remains to be demonstrated that similar patterns would be occurring in brain tissue and between different brain regions, recent findings provide some support in favour of blood as an acceptable proxy for brain methylation states^39–42^. Third, ours was by several standards a reductionist approach: in humans, CO_2_ hypersensitivity does not map PD with perfect specificity or sensitivity^3,5,12^, and in animals we used a single measure (tidal volume) to assess responses to hypercarbia. However, responses to the universally adversative stimulus of hypercarbia allowed for an interspecies’ comparative approach based on non-inferential laboratory readouts^12^. Moreover, changes in tidal volume are the key physiological strategy to reduce blood pCO2, and thus restore the physiological pH from the relative hypercapnia and acidification that ensue from breathing 6%-CO2-air mixture^2^. Fourth, in man, early-life adversities are associated with other phenotypes in addition to PD, such as anxiety, depression, or altered nociception, that are in turn often comorbid with PD. Therefore, when looking at our data, one might wonder about further potential links between RCF, our epigenetic findings, and other phenotypes. Indeed, altered nociception seems the most convincing example in this regard, given its association with RCF^25^, our findings with the asic1^25^ and asic2 (this study) genes, and the acidosis mechanisms shared between nociception, CO_2_ hypersensitivity, and fear^26,47^. Future studies with the RCF paradigm are needed to address these connections in a neurodevelopmental framework.

In conclusion, our findings indicate that epigenetic markers underlying CO_2_ hypersensitivity are shared between human and mouse, and part of these markers appear to be inherited across generations.

Future aetiological investigations may incorporate these findings and use such markers to better account for: the interplays between genetic and early-life environmental risk factors in anxiety disorders, the development of separation anxiety and panic disorder, and their familial transmission.

Future therapeutic investigations may consider intercepting the chemoreception mechanisms and brain pH dynamics that underlie CO_2_ hypersensitivity^48,53^ and panic attacks^2,9,10^ by targeting the *ASIC*s and related genes that our research is revealing as epigenetically enriched in humans and animals (this study^25^. These prospective studies could include ASIC antagonists such as amiloride, and encompass some RCF-associated phenotypes such as altered nociception^26^, which we showed to occur in RCF animals^25^ together with CO_2_ hypersensitivity^24,25^ and heightened separation anxiety indices^20,24^. Consistent with these findings, and evidence of the implication of the *ASICs* genes in specific human anxiety conditions^52,54^, children with high and persistent separation anxiety in preschool years^63^ go on to show altered respiratory (asthma reactivity) and nociceptive (headaches) physiology in mid-childhood and preadolescence^64^.

## Conclusions

We based this study on the well-known datum that people with panic and separation anxiety disorders overreact to brain-acidifying inhalation of CO_2_ by hyperventilating and panicking. We found that CO_2_ hypersensitivity can be experimentally induced by repeatedly cross-fostering new-born mice from their biological mother to other lactating females, and this is intergenerationally transmitted to normally-reared mice. The present study shows that CO_2_ hypersensitivity is associated with altered DNA methylation profile in both mice and man. Analyses of conserved mouse and human DNA methylation clusters revealed associations with morphogenesis and brain development, and with genes implicated in acid-sensing functions. Analysis of DNA methylation markers could provide a tool for prospective diagnostic applications.

## Methods

### Animal Rearing Protocols and the RCF Procedure

**Figure 5:**
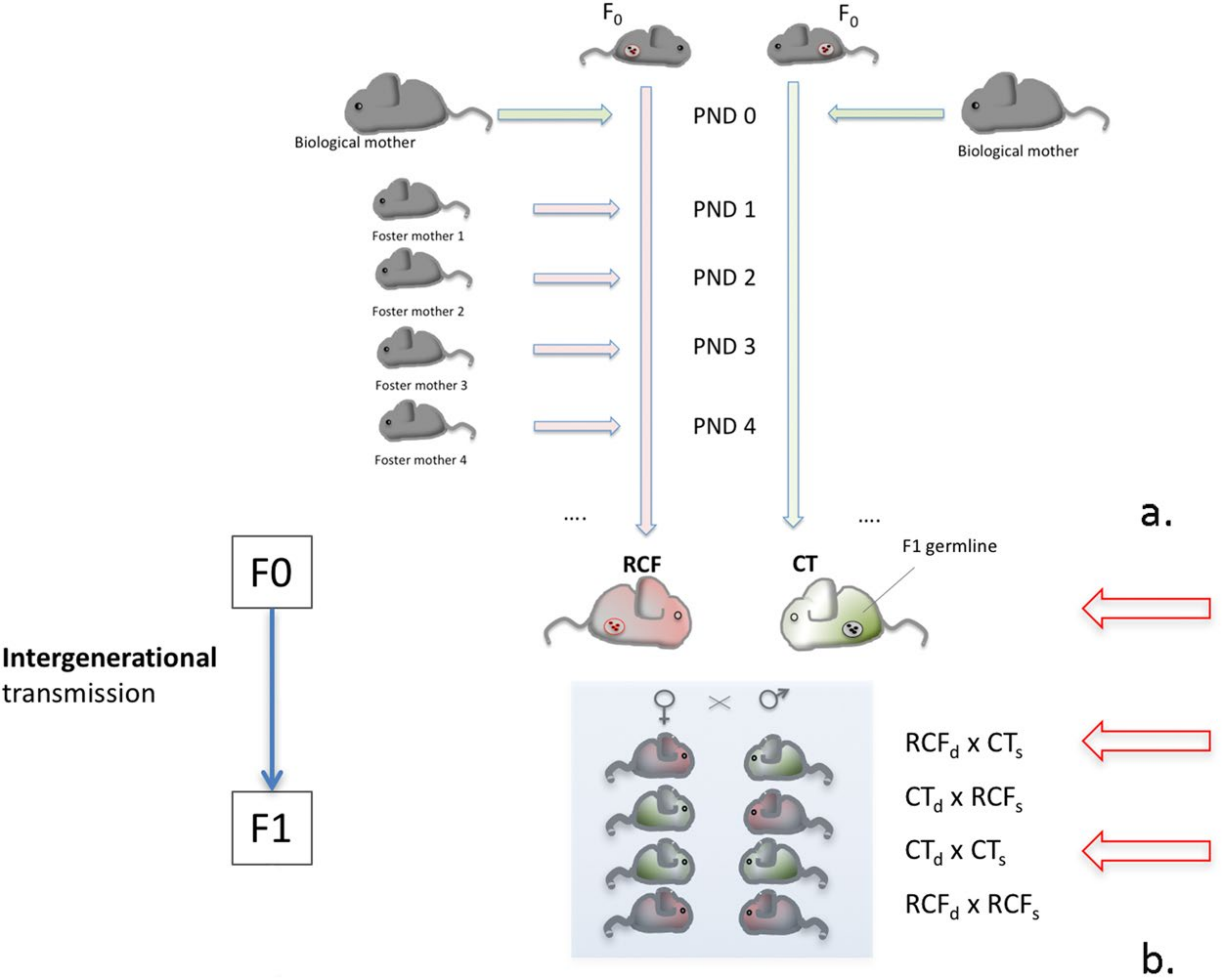
Graphical description of RCF experimental design. Top panel (a). RCF procedure (F0 generation); bottom panel (b). Parental crosses to obtain F1 animals. Red arrows depict experimental conditions selected for epigenetic analysis. PND, postnatal day; d, dams; s, sires.

For all experiments (conducted in agreement with- and under license DL 116/92 from the Italian Department of Health legislation on the use of animals for research, and the NIH guidelines) we employed outbred NMRI naïve mice (Harlan, Italy). The RCF is a cross-fostering procedure originally devised to interfere with newborn-mother relations in the first days of life, so to elicit offspring’s separation anxiety without inducing neglect from caregivers^20,24^. Having spent the first postnatal day (PND0) with the biological mother, on PND1 litters were culled to 8 pups (50% females) and randomly assigned to RCF^24^, or control (CT) treatment. Unlike more ‘classical’ cross- fostering procedures, RCF pups changed caregiver every 24 hours for 4 consecutive times in the PND1-PND4 time interval (Fig. 5a). By following a rotation scheme, each dam was shifted to 4 different litters and each litter was shifted to 4 different dams. The procedure of removing and cross- shifting a mother and the litter lasted about 30 seconds and was repeated daily, 4 times (PND1 to PND4), until reaching the fourth adoptive mother, with which pups remained until weaning. Adoptive dams were lactating females with pups of the same age as the fostered litters. Normally-reared control (CT) litters were collected daily and reintroduced to their home-cage, and had their biological mothers returned within 30 sec, from PND1 to PND4 (Fig. 5a; see also Supplementary Methods for details on the mating procedures). All animals took part in only one of the following experiments.

### Assessment of respiratory sensitivity to CO_2_

As in previous studies^20,24,25^ the association between RCF/CT rearing procedures and respiratory sensitivity to CO_2_ was assessed by measuring the percentage of increments in tidal volume (Δ%TV) during 6%CO_2_-enriched air vs. normal air breathing in an unrestrained plethysmograph (Buxco-PLY4211, Sharon CT), as described in details elsewhere^20,23,24^. Before any recording, each animal was acclimatized for 40 minutes. Then, the recording of respiratory parameters started under air condition (baseline) for 20 minutes. Next, a challenge began with the administration of 6%CO_2_-enriched air for 20 minutes, followed by a 20 minutes recovery period (air). Percentages of increment of tidal volume (TV) from the 20 minutes baseline air condition to 20 minutes 6%CO_2_ stimulation [Δ%TV] were compared between RCF vs. CT animals by general linear models (GLM), with sex factored in sex-balanced designs, or in post-hoc comparisons, as adequate.

### Assessment of intergenerational transmission of CO_2_ hypersensitivity from the RCF-exposed F0 parental generation to the normally-reared, non RCF-exposed F1 generation

We crossed (Fig. 5b): a) 10 unrelated dams that had been exposed as pups to RCF (F0-RCF_d_) to 10 unrelated sires that had been exposed as pups to RCF (F0-RCF_s_), b) 10 unrelated F0-RCF_d_ dams to 10 unrelated normally-reared (CT) sires (F0-CT_s_), c) 10 unrelated CT dams (F0-CT_d_) to 10 unrelated F0-RCF_s_, and d) 10 unrelated F0-CT_d_ to 10 unrelated F0-CT_s_. This yielded 33 litters in the following F1 generation (respectively litters: F1-RCF_d_RCF_s_: N = 9; F1-RCF_d_CT_s_: N = 8; F1-CT_d_RCF_s_: N = 7; F1-CT_d_CT_s_: N = 9), none of which had been exposed to RCF. All these F1 litters had been left with their biological mothers in normal rearing conditions while recording maternal behaviour. At PND 16–22, one/two pups/litter were chosen at random balancing for sex, to obtain 8 individuals for each of the 4 different parental crossings above [a), b), c), d), total 32 pups]. The Δ%TV responses during 6% CO_2_-enriched air vs. normal air breathing of these 32 F1 pups were analysed in a two-way ANOVA where the factors were: F0 maternal (RCF/CT) status, and F0 paternal (RCF/CT) status.

### Maternal Behaviour

As in our previous studies with the RCF^20,24,65^, maternal behaviour was observed daily from PND1 to PND7 in two sessions (12.00–12.30 and 16.00–16.30) by an observer unaware of the litter’s experimental group by instantaneous sampling (one sampling every 2 min, totalling 32 points/day/subject). The analyses of maternal behaviour in the intergenerational transmission of CO_2_ hypersensitivity were based on the evaluation of: a) NURSING (including the arched-back and blanket postures) and: b) grooming/licking (GP/L) behaviour on all 33 F1 litters, in 2 separate repeated measures ANOVAs. In both R-ANOVAs the independent factors were: 1) maternal early manipulation (2 levels: RCF, CT), 2) paternal early manipulation (2 levels: RCF, CT), and 3) the repeated variable factor, or days of observation (PND1–7).

Unless otherwise specified, animals were weaned when 28 days old, and then separated by sex and left in cages with littermates until sacrificed.

### Reproducibility of Intergenerational transmission of CO_2_ hypersensitivity

Having found evidence of maternal influence on the intergenerational transmission of CO_2_ hypersensitivity from F0 to F1 (see Results), to replicate the finding, we repeated a mating paradigm of crossing F0-RCF_d_ and F0-CT_d_ to normally-reared CT males for 3 different times in 3 different batches; this yielded 3 independent batches of normally-reared F1-RCF_d_ pups to be compared to F1-CT_d_ pups for replications of the respiratory responses to CO_2_. In these 3 replication samples the Δ%TV responses were analysed by ANOVA with the following factors: 1) maternal early-life manipulation (2 levels: RCF, CT), 2) batch (3 levels: batch 1, 2, or 3).

### Stability of CO_2_ hypersensitivity among F1-RCFd animals

To test CO_2_ hypersensitivity among F1-RCF_d_ animals in adulthood, we compared 26 naïve F1-RCF_d_ animals to 27 naïve F1-CT_d_ animals for their Δ%TV responses during 6% CO_2_-enriched air vs. normal air breathing at PND 75–90 by two-way ANOVA, with: F0 maternal treatment (RCF vs. CT) and sex (Males vs. Females) as factors.

### Assessment of CO_2_ hypersensitivity and identification of MZ Discordant Twin Pairs

Human participants were drawn from the largest human study of CO_2_ sensitivity published so far^14,15,20^ carried out in twins belonging in the Norwegian Institute of Public Health Twin Panel general population cohort (346 twin pairs, mean age: 30.95 ± 3.6 years, 49% MZ, 65% women). After complete description of the study, participants signed an informed consent and underwent an assessment of: lifetime DSM-IV psychiatric symptoms, lifetime adversities, and a CO_2_-respiratory challenge. This was a 35%CO_2_–65%O_2_ single-breath challenge, a safe, relatively non-invasive test that is extensively used at many academic institutions in Europe and North America, and discriminates well between patients with panic disorder, healthy subjects, or subjects with mental disorders other than panic^66^. Two gas mixtures were used: compressed air (placebo), and a mixture of 35%CO_2_–65%O_2_. Participants inhaled the gasses through a self-administration mask connected to a respirometer to measure vital capacity and the gas volume delivered at each inhalation; the test was considered valid if at least 80% of vital capacity was inhaled. Participants inhaled 1vital capacity of compressed air, and after an interval of 30 minutes, they inhaled 35%CO_2_–65%O_2_. At the end of each inhalation, participants held their breath for 4 seconds, and then rated the presence of symptoms on the Panic Symptom List III–Revised (PSL, that rates all 13 possible symptoms of a panic attack on a Likert scale, ranging 0 absent – 4 very intense each with total score range: 0–52), and the Visual Analog Scale for Anxiety (VASA, that rates anxiety between 0 -no anxiety at all- and 100 -the worst anxiety imaginable- on a visual continuous bar) obtained immediately after inhalation. By biometrical modelling of these outcome measures in this twin sample we showed that emotional reactivity to the 35%CO_2_−65%O_2_ challenge is substantially heritable (heritability ranging from 0.42 to 0.57). Among the 179 MZ pairs in the study, we identified 9 MZ pairs of women pairs as highly discordant (MZD) for CO_2_ sensitivity for having one twin with VASA and/or PSL response to challenge ≥90th percentile, and the other twin being a non-responder. Since human PD is more common in women^11^ and patterns of DNA methylation typically differ between males and females, DNA methylation analyses were limited to female animals and MZD women twin pairs. The 9 MZD women pairs (see Supplementary Table S11 for the main socio-demographic and clinical features, and Supplementary Table S12 for the VASA and PSL scores at the CO_2_ challenge) were matched to 9 randomly-selected age-matched MZ pairs of women concordant for absence (MZC) of any response to the CO_2_ challenge, to be employed as ‘controls’ to the MZD pairs. Genomic DNA from whole blood previously extracted with FlexiGene DNa kit (Qiagen) was used to assess the twins’ DNA methylation profiles.

### Animals for epigenetic DNA methylation experiments

Similarly to human samples, females were used for all the epigenetic experiments. We generated: a F0 batch of RCF and CT animals (F0), and their relative F1 normally-reared (F1) generation by following the same methods exposed above (Fig. 5b). Animals were sacrificed at PND 75–90, and brainstems were collected and kept at −80 °C until nucleic acid extraction^25^. The brainstems for the epigenetic experiments belonged to randomly-selected: 6 CT and 10 RCF females in F0, and 5 CT and 7 RCF females in F1.

DNA was extracted from frozen samples using MAXWELL16 automated extractor (Promega) using Maxwell16 Tissue DNA kit (Promega).

### Methylated DNA immunoprecipitation-seq (MeDIP-seq)

Four hundreds nanograms of genomic DNA were sheared with E220-COVARIS ultrasonicator (Covaris). Sequencing libraries were prepared with “NextFlex Methylseq kit 1” (Bioo Scientific). Immunoprecipitation was performed on ligated library using MagMeDIP (Diagenode) and purified using IPure kit (Diagenode). For mouse samples, groups of 8 randomly-selected libraries were pooled and immunoprecipitated in the same reaction to reduce technical biases. Paired-end sequencing was performed on the Illumina HiSeq2500 platform with read length of 100 bp.

### Analysis of meDIP-seq data

Number of reads, distribution of CG percentage within reads, number of duplicated reads were assessed with the FastQC software 0.10. 1^67^. Reads were mapped to the reference genome, version hg19 for human data, and mm10 for mouse data, with the Burrows-Wheeler Aligner^68^. Only uniquely aligned and properly paired read tags with mapping score >15 were retained for subsequent analysis. (Supplementary Tables S13 and S14). Methylated regions were identified with MACS2^69^. Regions obtained from different samples were merged by the bedtools suite^66^ and statistical analyses were performed on the matrix of read counts over all regions. Data were normalized using Trimmed Mean of M-values (TMM^70^), and analysed with R 3.1.1^71^. After identification DMRs were annotated as described in Supplementary Methods.

### Human DMRs identification

The logarithm of fold change (logFC) was computed by comparing the normalized counts of the responder vs. non-responder twin within each MZD pair. A linear model was then applied to logFCs by the “lmFit” function of the limma 3.22.7 package from Bioconductor^72^ and DMRs were retained with a p threshold = 0.001. For the MZCs, an absolute value of logFC was computed for the intra-pair comparison over the methylation peaks of MZDs. The absolute values of logFCs were then compared between MZD and MZC samples by one-tail Wilcoxon test.

### Animal DMRs identification

For the mouse experiment (F0, F1 generations) regions with a number of counts per million (cpm) >1 in at least N = 5 samples (i.e. the size of the smallest experimental group) were selected for analysis. A generalized linear model was applied to the matrix of normalized counts to identify DMRs between RCF and CT experiment-wise, including the information about the immunoprecipitation pools (N = 6) as factor, by the “glmFit” function from 3.22.7 edgeR. Regions with p-value < 0.001 were selected for further analyses.

### DNA methylation cluster analysis

DNA methylation clusters were identified by Density Based Spatial Clustering of Applications via the Noise-DBSCAN algorithm^73^. A distance cut-off (ε) of 10′000 bp was used, with a minimum number of points required to form a dense region (*n*) = 2. ccDMRs (*p*-value < 0.001) were selected for the subsequent analyses.

### Annotation of clusters of methylated regions

Clusters were annotated with overlapping genes and genes located within 100 kb, either upstream or downstream. This cut-off was chosen taking into account the average size of chromatin contact domains, defined as regions with the highest probability of establishing contacts with other loci. Those regions have been shown to range from 10^4^ to 10^6^ bp^74,75^. Association of gene list to Gene Ontology (GO) terms was performed using the GOStats package^76^ and hypergeometric-based test. The derived gene background set consisted of genes associated with any clusters. Since genes included in the same ccDMR can be functionally related^31,32^. Fisher’s exact test could be biased and result in overly inflated p-values. To tackle this bias, we randomly extracted one gene from each cluster to perform GO enrichment analysis. These randomizations were reiterated 100 times, and the geometric mean of GO term ranks (ordered by ascending p-value) was used to score results. To estimate an empirical p-value for the enrichment results we also performed 1000 shuffling of the abovementioned scores.

### Semantic similarity analysis

For each ontology (Biological process: BP; Cellular component: CC; Molecular function: MF), the semantic similarity of the top 50 GO terms was computed, after filtering for *p*-value < 0.01 and a number of associated genes >1 and <50 was identified and visualised with the ReviGO webtool (http://revigo.irb.hr/)^77^. The GO term number and ranking score were used as the input.

### DNA methylation clusters, Human-mouse comparison

Intersections between sets of clusters were computed with the “intersectBed” tool from the Bedtools suite^69^. In order to assess the cross-species consistency of methylation clusters, human cluster coordinates (hg19 assembly) were mapped to orthologous mouse coordinates (mm10 assembly) with the UCSC “LiftOver” tool (“minMatch” = 0.1). Estimation of Tissue-specific chromatin state feature enrichment was performed as detailed in Supplementary Methods.

### Ethics approval and consent to participate

The procedure of the 35% CO2 -challenge was approved by the Regional Committee for Medical Research Ethics, authorized by the Norwegian Government.

All animal experiments were conducted under license from the Italian Health Department and in accordance with Italian regulations on the use of research animals (legislation DL 116/92) and NIH guidelines on animal care.

### Availability of data and material

The datasets used and/or analysed during the current study are available from the corresponding author on reasonable request^78^.

## Electronic supplementary material

Supplementary Files
