# Supplementary material for: Conserved DNA Methylation Signatures in Early Maternal Separation and in Twins Discordant for CO2 Sensitivity
Source: Sci Rep. 2018 Feb 2;8:2258. doi: 10.1038/s41598-018-20457-3 (PMC5797081; doi:10.1038/s41598-018-20457-3)
Supplement: Supplementary file 1 — Supplementary Files [file 41598_2018_20457_MOESM1_ESM.pdf]

Supplementary Files

Conserved DNA Methylation Signatures in Early Maternal Separation and in Twins Discordant for CO_2 Sensitivity

Francesca Giannese, Alessandra Luchetti, Giulia Barbiera, Valentina Lampis, Claudio Zanettini, Gun Peggy Knudsen, Simona Scaini, Dejan Lazarevic, Davide Cittaro, Francesca R. D'Amato, and Marco Battaglia.

Supplementary Materials and Methods

Supplementary Figures

Supplementary Tables

Supplementary Materials and Methods

Mating Protocol

Mice were mated when they were 12 weeks old. Mating protocol consisted in housing two females with one male in transparent high temperature polysufone cages (26.7 x 20.7 x 14.0 cm) with water and food available *ad libitum*. Room temperature (21$\pm$ 1°C) and a 12:12 h light dark cycle (lights on at 07.00 p.m.) were kept constant. After 15 days males were removed and pregnant females were isolated, left in clean cages, and inspected twice a day for live pups.

Annotation of DMRs

DMRs were associated with genes by ChIPpeakAnno [1] and GENCODE gene model (version M9 for mouse data and version 19 for human data). Intragenic DMRs were associated with the overlapping gene, while intergenic DMRs were associated with the gene with the closest transcription start site (TSS). The resulting gene list was used to perform functional enrichment with the Enrichr platform [2].

To analyse enrichments for specific genomic features, the DMRs were annotated to: promoters (defined as spanning -1000 to +1000 from gene TSS), exons, introns, CpG islands, 3'-UTRs, and 5'-UTRs by using GENCODE gene models, repetitive elements (simple – single nucleotide stretches and tandem repeats, Satellite repeats, Short Interspersed Nucleotide Elements – SINE-, Long Interspersed Nucleotide Elements – LINE-, long terminal repeats – LTR-, Low Complexity DNA, RNA repeats and DNA transposon classes) and super-enhancers. Bed files corresponding to each feature were downloaded from UCSC. Super-enhancers definitions were obtained from the dbSUPER database (http://bioinfo.au.tsinghua.edu.cn/dbsuper/)[3].

For human data analyses, brain- and blood-specific dataset were obtained by merging coordinates from all available tissue-specific datasets via the “mergeBed” command available in bedtools [4]. For mouse data annotation, the E14.5 brain tissue dataset was used, after converting coordinates to the mm10 genome assembly via the LiftOver tool from UCSC.

DMRs were also annotated to 15-state chromatin models as defined by the Roadmap Epigenomics Consortium [5]. Specifically, datasets relative to tissue groups: “Blood & T-cell” and “HSC & B-cell” (Epigenome Order ID 24 to 46), and “Brain” (Epigenome Order ID 64 to 73) were selected for blood- and brain tissues respectively. For each 15-chromatin state, we created a tissue meta-state by merging the intervals defined in brain, or blood files separately.

The chromatin states definition for mouse brain were obtained from publicly released data (https://github.com/gireeshkbogu/chromatin_states_chromHMM_mm9) [6]; coordinates were converted to mm10 assembly via the “LiftOver” tool from UCSC. According to state functional annotation, the 15-state model was reduced to a 7-state model: states 12 to 14 were merged within a single “Heterochromatin” state; states 5 and 7 were merged within “Active Promoter” state, states 6 and 8 were merged within “Strong enhancer”, 9 and 4 within “Poised enhancer” and 1,2,3 within “Transcribed” state, as described in Bogu et al. [6].

The number of DMRs associated with each feature was computed with the “interesectBed” tool available in Bedtools [4]. To obtain an empirical *p*-value of the observed intersections, we randomly shuffled DMRs coordinates with the “shuffleBed” routine over clusters of methylated regions, as specified in the next paragraph; we avoided shuffling over the entire genome to prevent overestimating statistical significance. Shuffling was reiterated 1000 times. Enrichment *p*-values were computed by comparing the expected occurrence value with the observed value in the dataset.

Tissue specific chromatin state feature enrichment

To analyse the tissue specificity of chromatin states for DNA methylation clusters, we downloaded the core chromatin state annotations (15 states for 127 cell lines) from the Roadmap Epigenome data [5], see also **Supplementary Table S15**)

We first defined the coverage '*C'* of each chromatin state '*s'* (E1 to E15) on human genome in its specific cell type '*t'* as the coverage of all the '*i'* over the effective size of the human genome (*i.e.* the genome fraction that can be sequenced):

$$C_{s,t} = \sum_i \frac{l_i}{2.7 \cdot 10^9}$$

Resulting in a 15x127 matrix.

Then, for each methylation cluster *m* (only conserved clusters were considered), we calculated the overlap *o* with the cluster and calculated the ratio C_m

$$C_{m,s,t} = \frac{\sum o_{s,t}}{l_m}$$

again in a tissue specific manner, resulting in 35 15x127 matrices.

Each matrix was summarized row-wise (*i.e.* values for each chromatin state) by averaging the values pertaining to the same tissue (Brain, Smooth Muscle, Muscle, Heart, Digestive, Adipose, Mesenchymal, Epithelial, Thymus, Blood & T-cell, HSC & B-cell and ESC) according to **Supplementary Table S15.** After this process, we obtained 1 background and 35 cluster-specific (M_i) matrices, representing tissues (*B*), of 15x12 each. We evaluated the normalized ratio *E* of each state annotation in each tissue as

$$E_i = \frac{M_i}{B}$$

Distances between two (*x, y*) clusters were calculated as

$$D = \left\| E_x - E_y \right\|$$

This distance was used to perform agglomerative clustering via the Ward method [7].

The matrices defining each cluster were also used to assess tissue specificity. Briefly, each matrix had 15 rows corresponding to chromatin states, and 12 columns corresponding to different tissues. For each column we calculated the correlation with a vector

$$v = [1, 1, 1, 1, 1, 1, 1, -1, -1, -1, -1, -1, -1, -1, -1]$$

with a value = 1 for chromatin activation states, and value = -1 for chromatin repression states. Hence, each tissue received a positive correlation value if state was activated, and a negative correlation value if state was repressed. Tissues and clusters were matched on the basis of the highest correlation score.

References

1. Zhu, L. J. *et al*. ChIPpeakAnno: a Bioconductor package to annotate ChIP-seq and ChIP-chip data. *BMC Bioinformatics* **11**, 1 (2010).

2. Chen, E. Y. *et al*. Enrichr: interactive and collaborative HTML5 gene list enrichment analysis tool. *BMC Bioinformatics* **14**, 128-2105-14-128 (2013).

3. Khan, A. & Zhang, X. dbSUPER: a database of super-enhancers in mouse and human genome. *Nucleic Acids Res.*, gkv1002 (2015).

4. Quinlan, A. R. BEDTools: the Swiss army tool for genome feature analysis. *Current protocols in bioinformatics*, 11.12.1-11.12.34 (2014).

5. Kundaje, A. *et al*. Integrative analysis of 111 reference human epigenomes. *Nature* **518**, 317-330 (2015).

6. Bogu, G. K. *et al*. Chromatin and RNA Maps Reveal Regulatory Long Noncoding RNAs in Mouse. *Mol. Cell. Biol.* **36**, 809-819 (2015).

7. Ward Jr, J. H. Hierarchical grouping to optimize an objective function. *Journal of the American statistical association* **58**, 236-244 (1963).

Supplementary Figures

Supplementary Figure S1
Maternal cares (nursing behaviour and grooming/licking behaviour) received by F1 pups.
Repeated measures ANOVA applied to 33 litters (see also Methods section) showed that neither F0- maternal nor paternal early-life treatment (RCF vs CT), or their interaction, exerted a significant effect on nursing. Only time ($F_{6,174}$= 9.11, p< 0.0001) had a significant effect, reflecting the physiological reduction of nursing from post-natal day (PND) 1 to PND 7, without significant interaction with any of the other aforementioned factors in the R-ANOVA.
Similarly, repeated measures ANOVA on grooming/licking behaviour (bottom of figure) showed no effect of: F0-maternal, or F0-paternal early-life treatment (RCF vs. CT), time, or any of their interactions. Paternal F0 early rearing experience (RCF, or CT), and its interaction with maternal RCF/CT status (not shown in Figure for the sake of simplicity), also resulted in non-significant differences in nursing and licking/grooming received by F1 pups.

Supplementary Figure S2:
Consistency of respiratory responses across different F1 animal batches.
Responses to 6%CO_2-enriched air vs. normal air at PND 16-22 among F1-RCFd pups (born to F0 dams that had experienced RCF mated with normally-reared sires) and F1-CTd pups (born to normally-reared F0 dams mated with normally-reared sires) across 3 batches. ANOVA showed a significant difference between F1-RCFd and F1-CTd for Δ%TV responses to 6% CO2-enriched air ($F_{2,67}$=8.44, p<0.005). As expected with outbred animals belonging to 3 different batches, there was a significant 'batch' variation effect on respiratory responses ($F_{2,67}$=3.97, p<0.05), but no significant interaction between 'batch' and F0 lineage ($F_{2,67}$=0.29 p=0.74; see also Supplementary Table 1 for breakdown of N, and values of responses across the 3 different batches of F1 pups).

Supplementary Figure S3: CO_2 hypersensitivity in adult naïve F1 animals.

The Δ%TV responses to 6% CO2-enriched air were significantly higher amongst normally-reared F1-RCFd adult (PND 75-90) naïve normally-reared animals whose biological mothers had experienced RCF, than those of F1-CTd adult naïve normally-reared animals whose biological mothers had not experienced RCF (F1-RCFd: 42.22+3.53 vs. F1-CTd: 28.08+2.67; $F_{1,49}$=9.38, p<0.04). Neither sex ($F_{1,49}$=0.39, p=NS) nor the interaction between sex and maternal F0 treatment ($F_{1,49}$=0.25, p=NS) significantly influenced these responses.

Supplementary Figure S4. Associations of DMRs with genomic features across F0 and F1 mouse experiments (RCF exposed vs. normally reared animals).
The expected number of DMR-associated features was obtained by randomising DMRs genomic intervals over DNA methylation clusters; $\log_{10}$ *p*-values were computed by comparing the number of expected vs. observed DMRs for each genomic feature. Red bars indicate p=0.01 threshold

Supplementary Figure S5. Semantic Similarity for GO terms associated with genes in F0 (panel a) and F1(panel b) methylation clusters (RCF exposed vs. normally reared animals).
Each circle symbolizes a GO term; circle size is proportional to term frequency (greater size indicates a more general term). Circle colour indicates term uniqueness (divergence from other terms) and label colour indicates dispensability (black: dispensability<0.15). Term relevance was computed by GO list ranking. Scatterplot elaborated using ReviGO (revigo.irb.hr).

Supplementary Figure S6 Associations of DMRs with genomic features in human MZ twins discordant for CO_2 hypersensitivity.
The expected number of DMR-associated features was obtained by randomising DMRs genomic intervals over DNA methylation clusters; $\log_{10}$ *p*-values were computed by comparing the number of expected vs. observed DMRs for each genomic feature. Positive values indicate enrichment, and negative values indicate depletion. The red line marks a p-value threshold =0.01.

Supplementary Figure S7. Semantic Similarity for GO Terms Associated with genes in MZ Twins Methylation Clusters.
Each circle symbolizes a GO term; circle size is proportional to term frequency (greater size indicates a more general term). Circle colour indicates term uniqueness (divergence from other terms) and label colour indicates dispensability (black: dispensability<0.15). Term relevance was computed by GO list ranking. Scatterplot elaborated by ReviGO (revigo.irb.hr).

Supplementary Figure S8. **Hierarchical clustering analysis: Dendrogram by chromatin state annotation**
Human MZ methylation clusters grouped by amount of enrichment of tissue-specific chromatin states.Green, group 1; red, group 2, cyan, group 3; violet, group 4

Supplementary Figure S9. Heatmaps representing the relative chromatin state frequency (fold enrichment) for each Group 1 cluster

Each column represents a reference epigenome (brain, smooth muscle, muscle, heart, digestive, adipose, mesenchimal, epithelial, thymus, blood and T-cell, HSC and B-cells, embryonic stem cells)

Supplementary Figure S10. MZ twins DNA methylation signals

Detail for Cluster 46, and *asic2*-associated DMR

Bigwig tracks for both discordant and concordant twins are shown superimposed. Profiles of twins with positive response to a CO_2 challenge are shown in red, while non-responders' profiles are in green. Profiles of twins concordant for absence of response CO_2 challenge are in blue.

Supplementary Figure S1

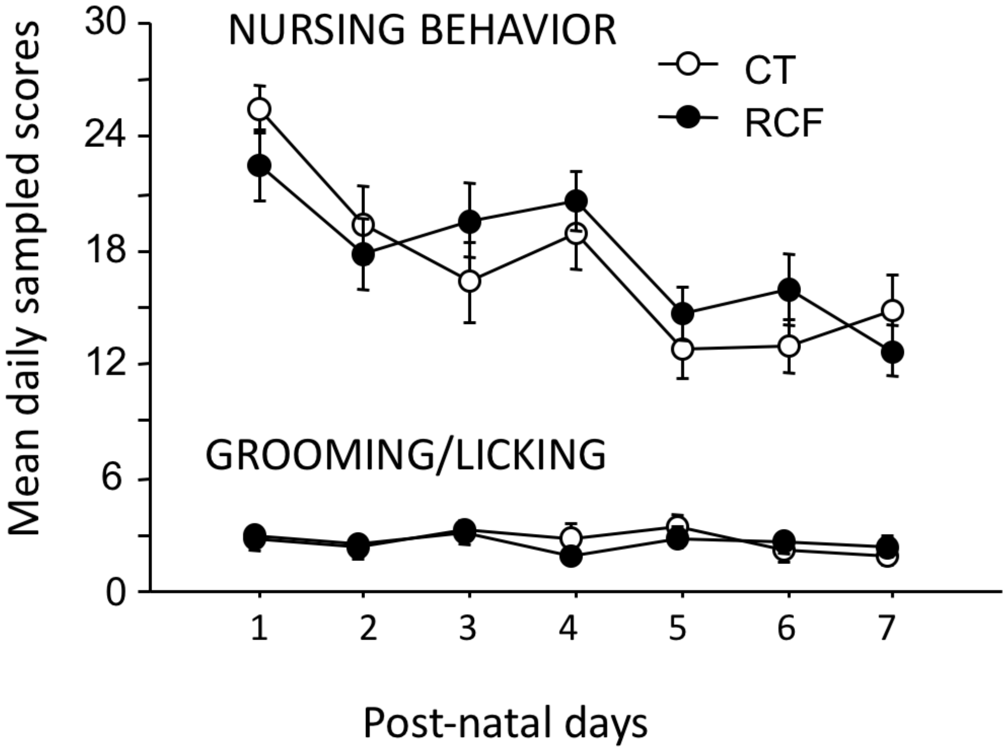

Supplementary Figure S2

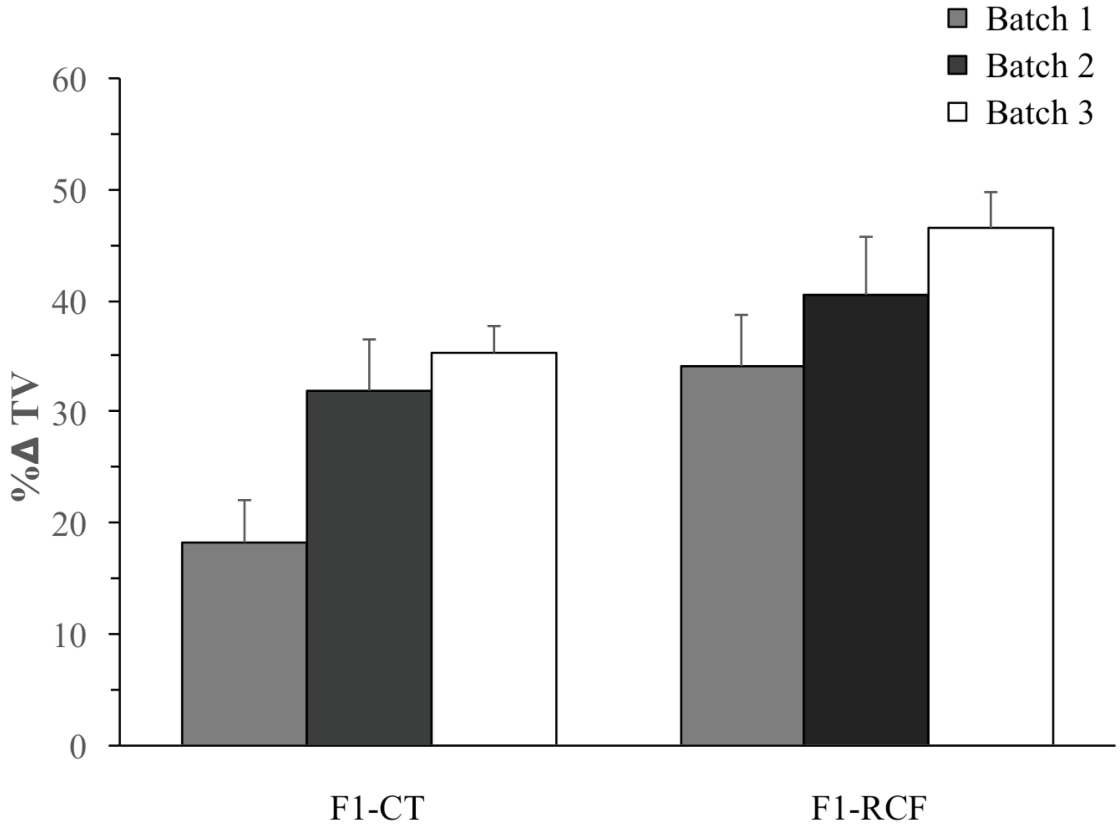

Supplementary Figure S3

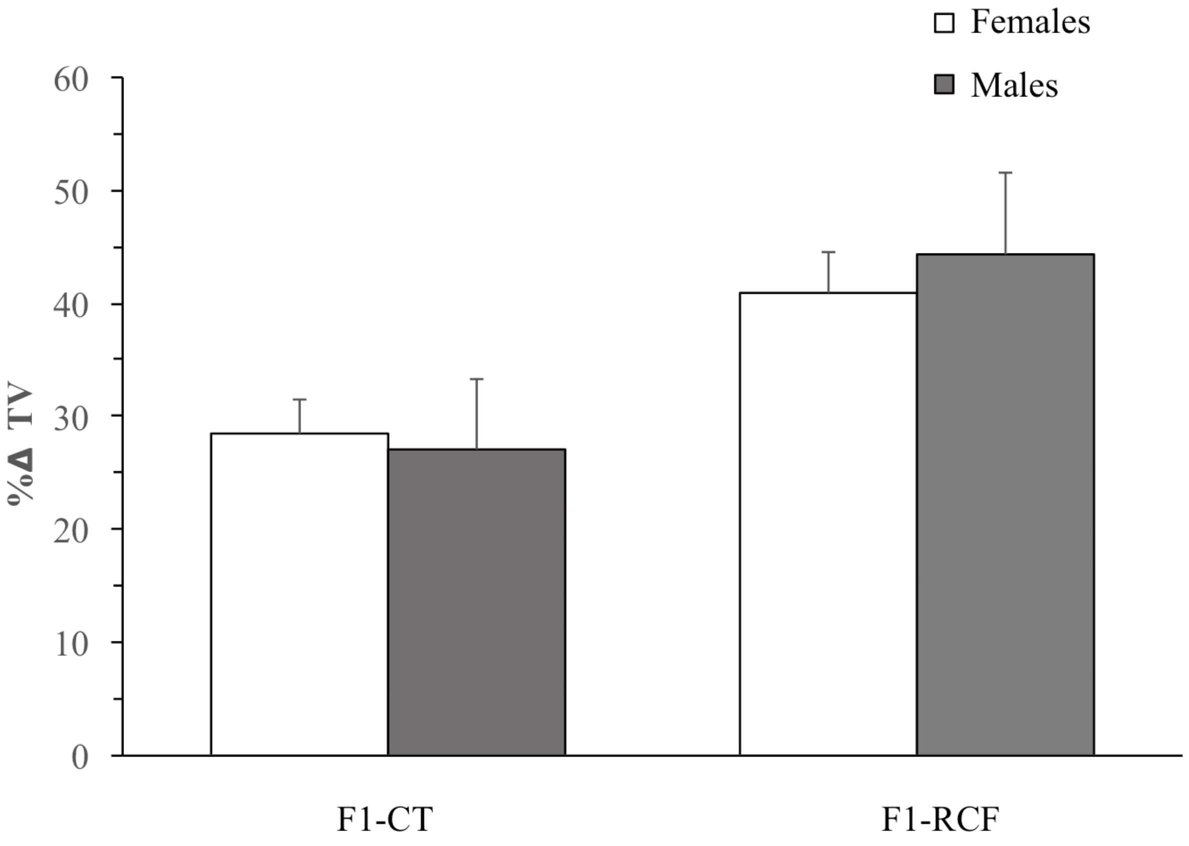

Supplementary Figure S4

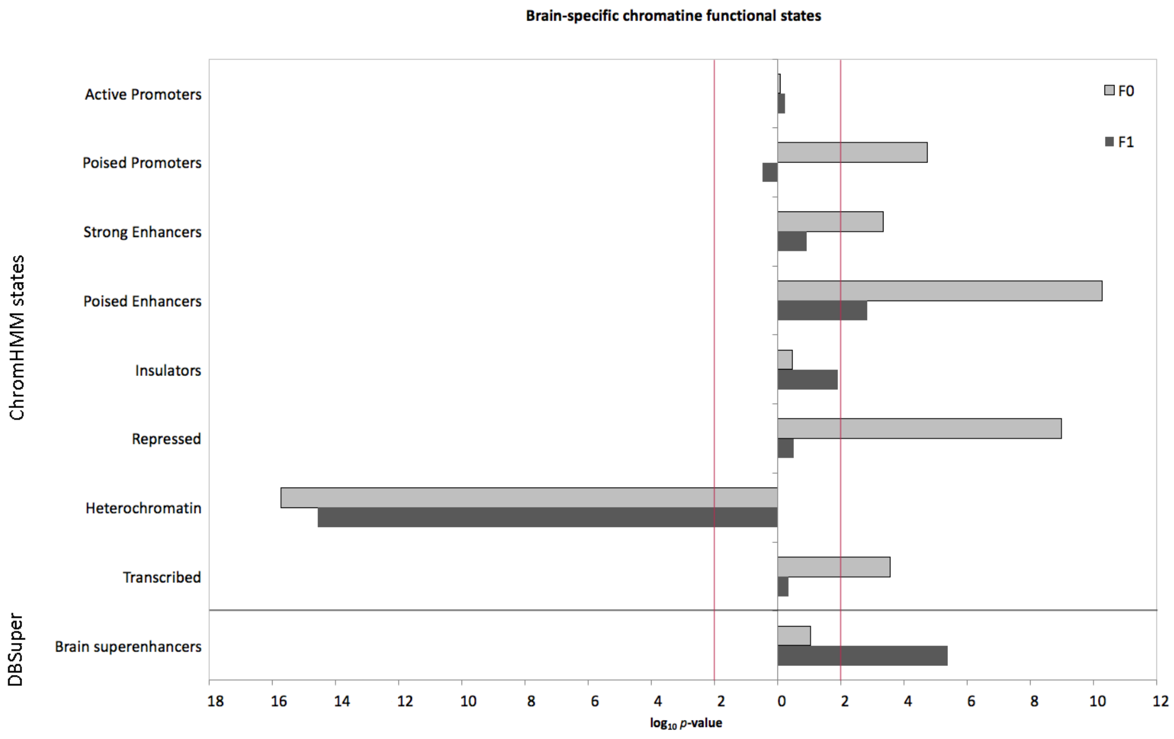

a.

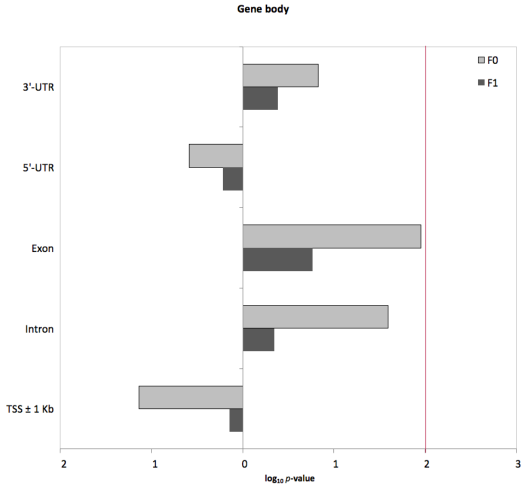

b.

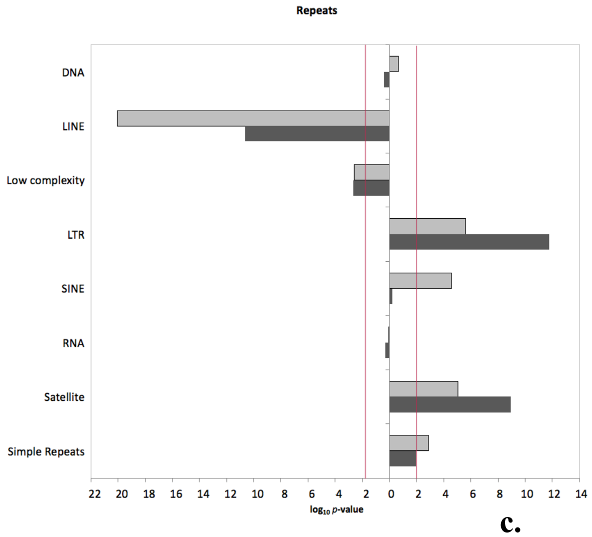

c.

Supplementary Figure S5

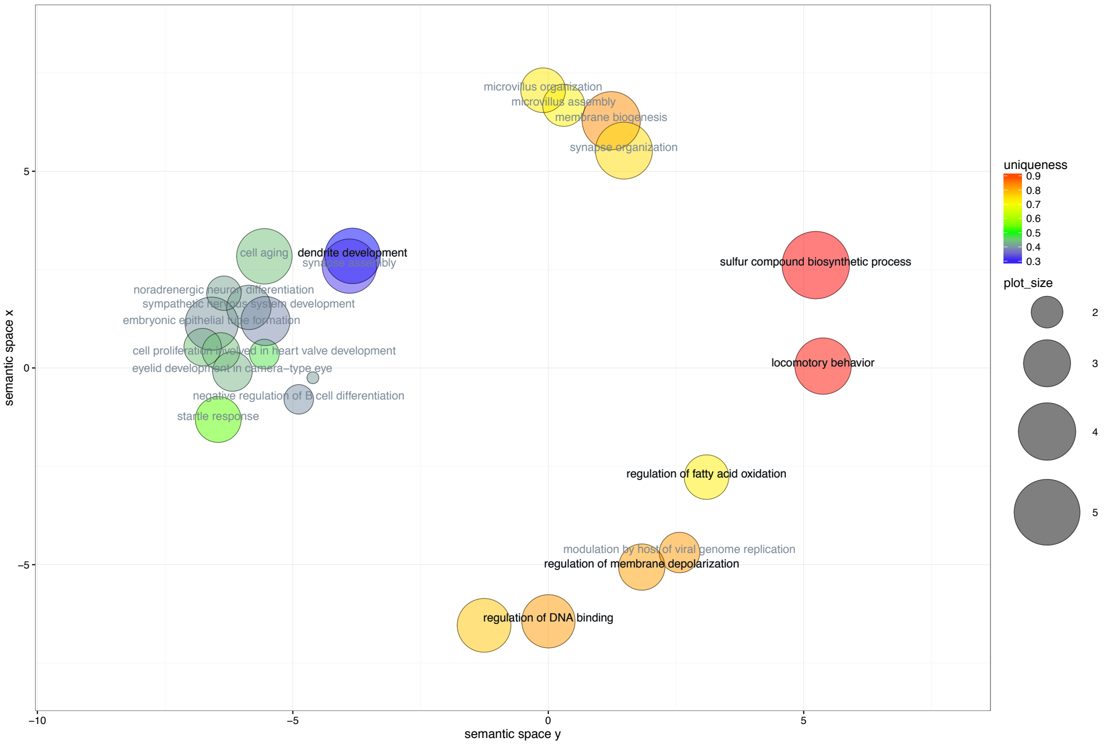

a.

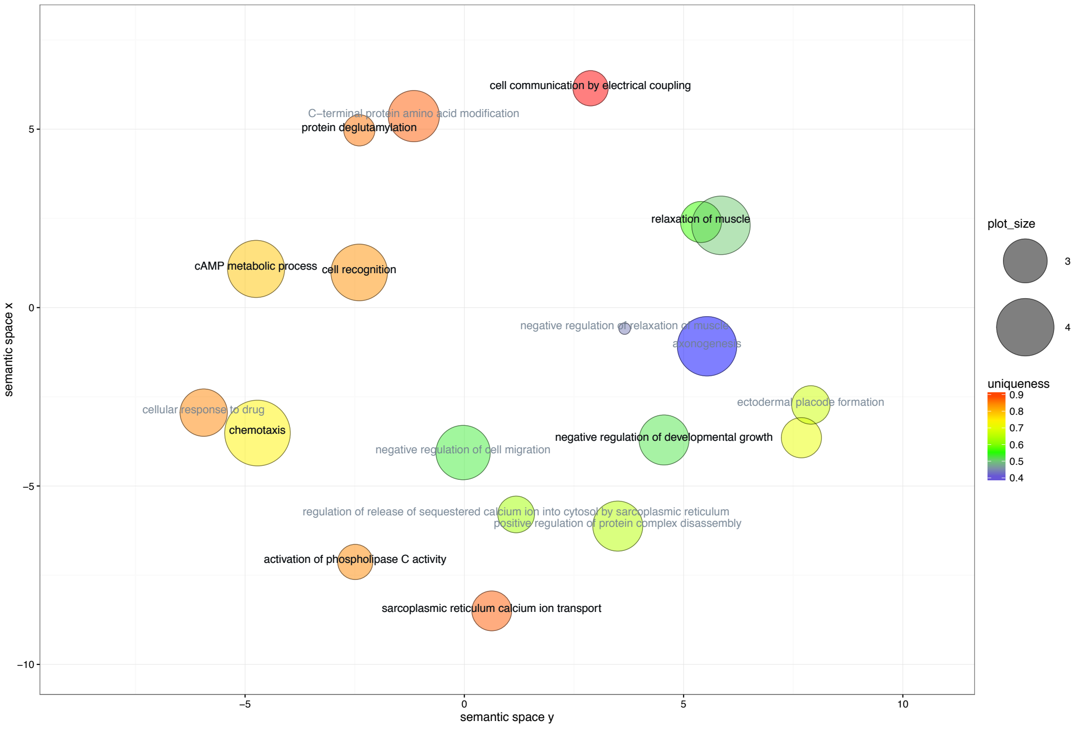

b.

Supplementary Figure S6

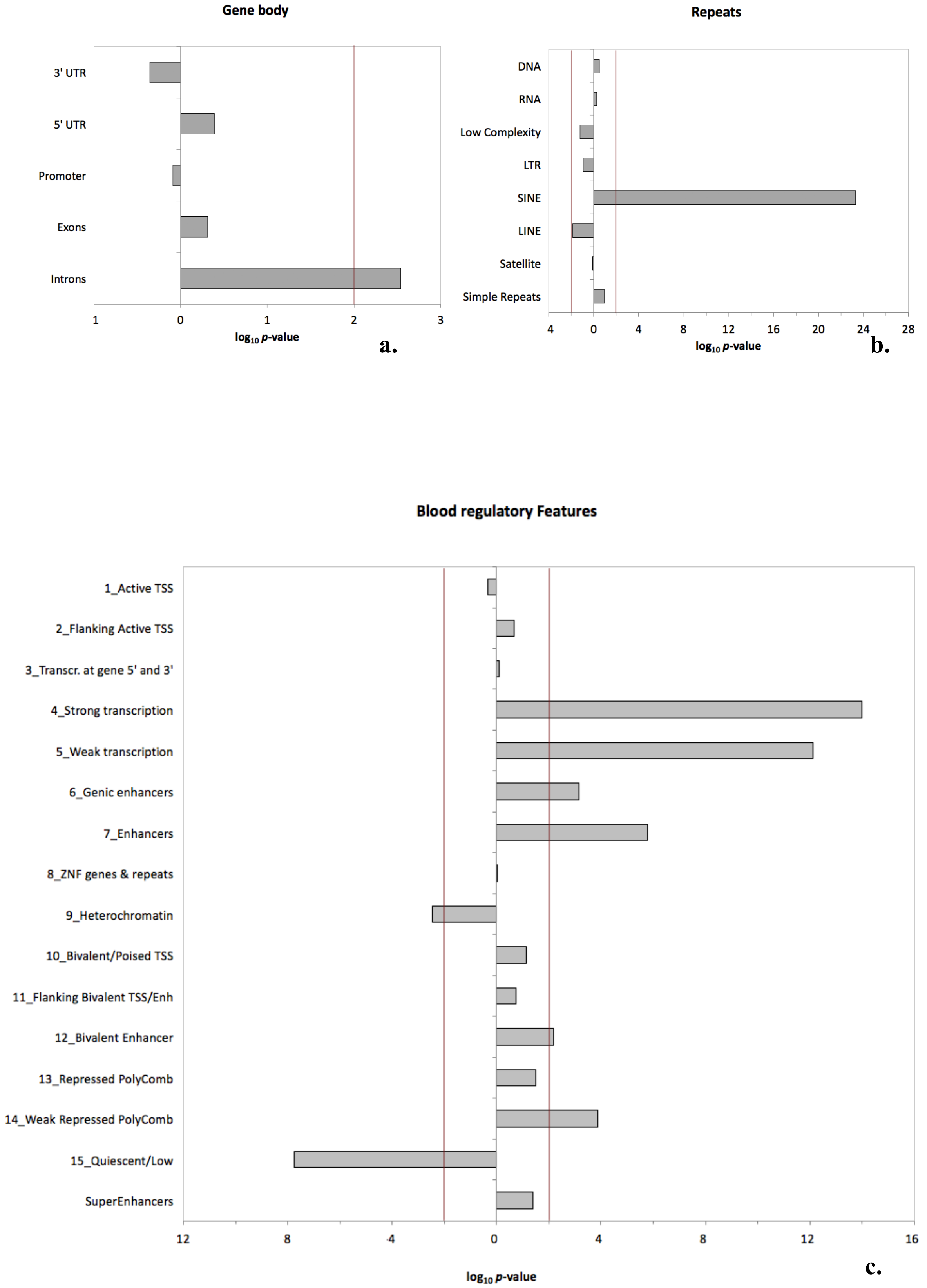

Supplementary Figure S7

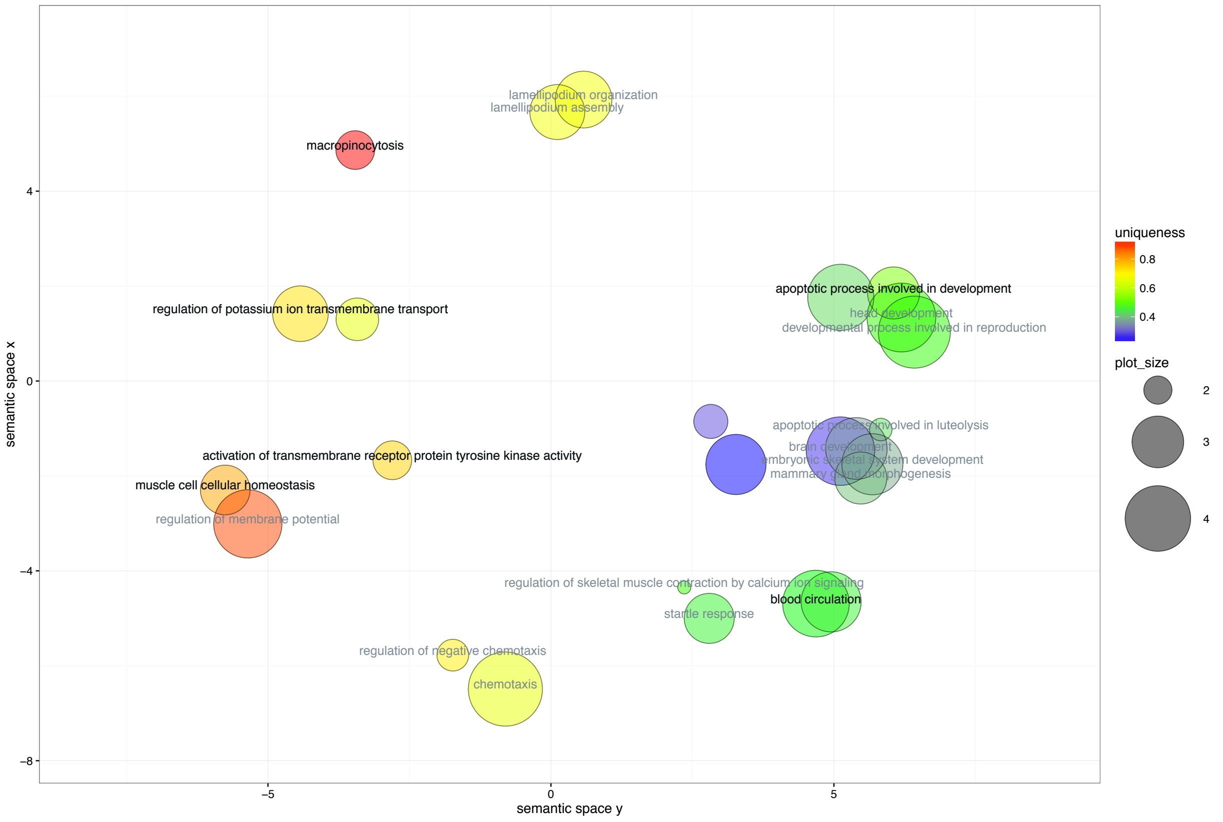

Supplementary Figure S8

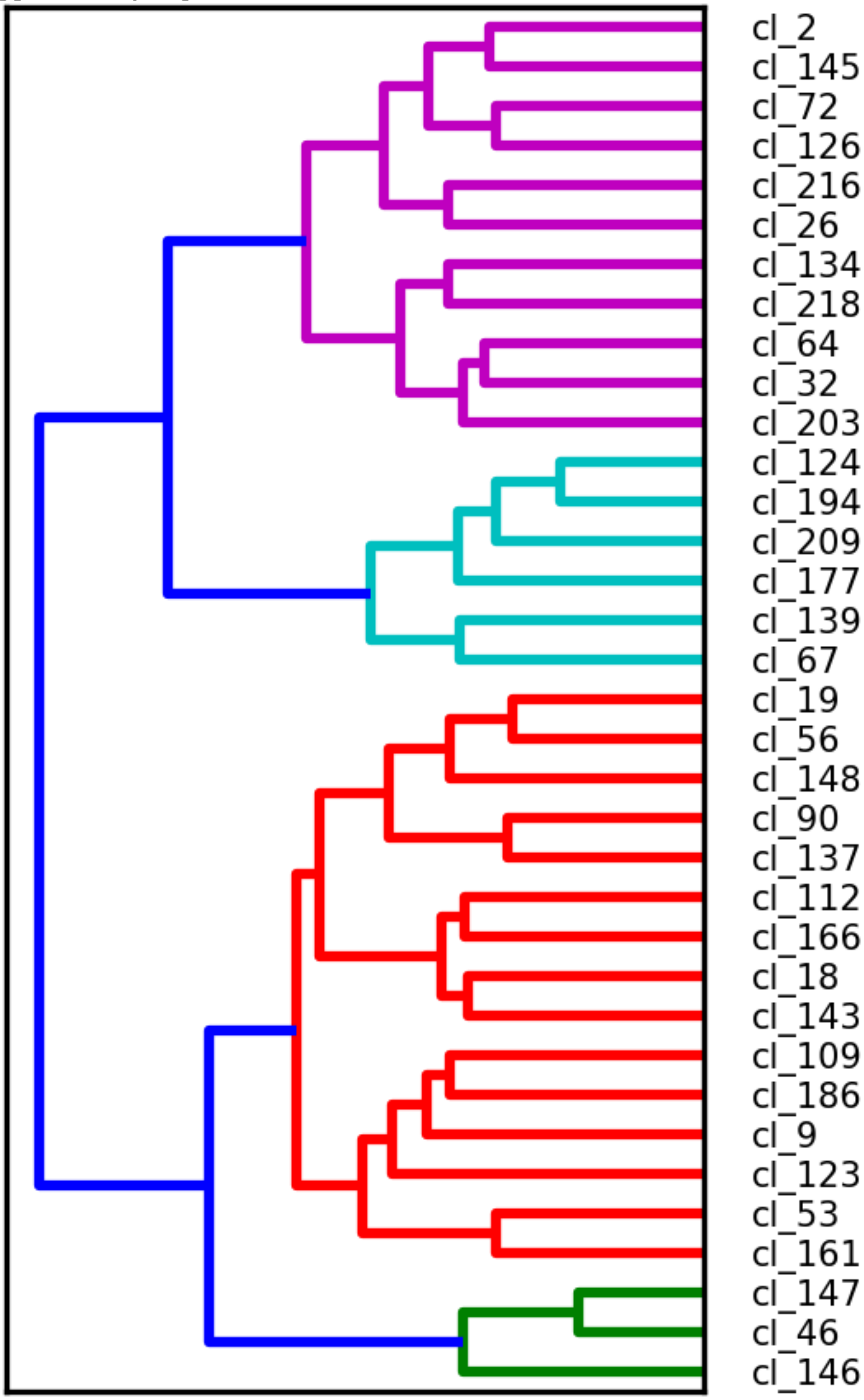

Supplementary Figure S9

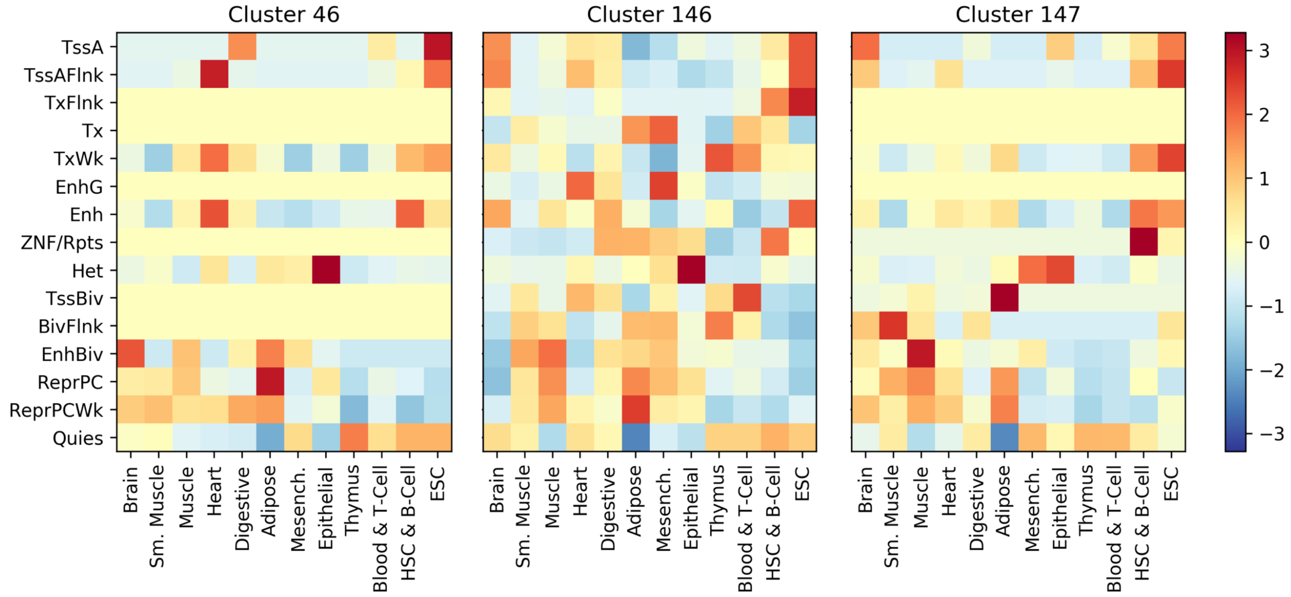

Supplementary Figure S10

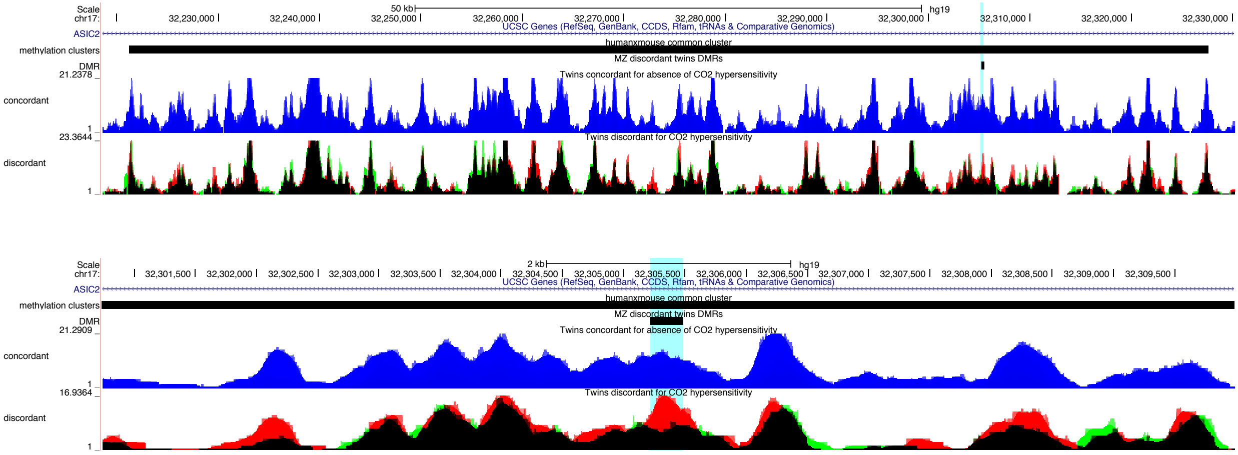

Supplementary Tables

Supplementary Table S1 : Breakdown of respiratory responses (mean Δ%TV under 6%CO_2) in normally-reared F1 pups belonging to 3 different batches, divided by maternal postnatal treatment. F1-RCF_d indicates normally-reared pups born to mothers that had experienced RCF, mated with CT (normally-reared) sires; F1-CT_d indicates normally-reared pups born to CT mothers mated to CT sires.

Maternal Treatment: CT		N	Mean	SE	Maternal Treatment: RCF		N	Mean	SE
F1-CT_d	Batch 1	8	18.29	3.71	F1-RCF_d	Batch 1	14	34.13	4.57
	Batch 2	15	31.82	4.59		Batch 2	18	40.45	5.27
	Batch 3	8	35.25	2.51		Batch 3	10	46.64	3.20

Supplementary table S2. MACS2 peak calling and results of differential methylation analyses contrasting the CT vs. RCF animals

**Experiment**	**Number of peaks**	**DMR (p-value<0.001)**		
		total	*hyper-methylated*	*hypo-methylated*
**F0**	566067	976	555	421
**F1**		759	477	282

Supplementary Table S3. DNA methylation clusters details for mouse F0/F1 experiments.

**Experiment**	**Number of clusters**	**Clusters containing DMRs with p-value<0.001 (ccDMR)**	**Number of associated genes**	**ccDMR Median size (bp)**
**F0**	44054	779	4548	91708
**F1**		575	3189	87185

Supplementary Table S4. Top 50 Biological Processes Gene Ontology terms associated with mouse F0 methylation clusters. Table reports terms (p-value<0.01) selected after filtering for a number of associated genes >1 and < 50 and ranked according to geometrical mean of ranks in 100 randomizations.

GO ID	Term	Rank product	Associated genes
GO:0016358	dendrite development	34	Btbd3,Cit,Cpeb3,Dlg4,Lst1,Palm,Ptprd,Rac1,Sdk1,Grip1,Abl2,Dab1,Fmn1,Abi2,Cux2,Dscam,Elavl4,Fstl4,Fyn,Grin3a,Klhl1,Map1b,Nedd4,Nrg1,Pten,Slc11a2,Syngap1,Camk1,Celsr2,Ctnnd2,Cyth2,Farp1,Fbxo31,Id1,Kalrn,Nck2,Numbl,Plk2,Prmt3,Rap2a,Rapgef4,Sema4d,Sipa1l1,Syne1
GO:0048813	dendrite morphogenesis	89	Btbd3,Cit,Dlg4,Ptprd,Abl2,Fmn1,Rac1,Cux2,Dscam,Elavl4,Fyn,Nedd4,Pten,Slc11a2,Celsr2,Ctnnd2,Farp1,Fbxo31,Id1,Numbl,Prmt3,Rap2a,Sema4d,Sipa1l1,Syne1
GO:0021840	directional guidance of interneurons involved in migration from the subpallium to the cortex	95	Nrg1,Nrg3
GO:0021842	chemorepulsion involved in interneuron migration from the subpallium to the cortex	98	Nrg1,Nrg3
GO:0007416	synapse assembly	115	Adgrb1,Cbln1,Lrtm2,Ptprd,Syndig1,Tpbg,Nrxn3,Clstn2,Asic2,Cux2,Nrg1,Pdgfb,Pten,Sdk2,Adnp,Cdh1,Farp1,Thbs2
GO:0007626	locomotory behavior	119	Qrfp,Cacna1c,Hexb,Lmx1a,Npc1,Pak6,Rcan2,Sez6l,Abhd12,Adra1b,Aldh1a3,Cacnb4,Cdh23,Cln8,Dab1,Dbh,Dlg4,Dscam,Elavl4,Espn,Etv5,Gaa,Gmfb,Grin2d,Id2,Klhl1,Lsamp,Mc3r,Nav2,Npas2,Npas3,Nrg1,Ntf5,Olfm2,Otog,Park2,Pten,Scn8a,Sptbn4,Tmod1,Tshr,Zfhx3,Cxcl12
GO:0050808	synapse organization	167	Adgrb1,Cbln1,Gdnf,Lrtm2,Palm,Ptprd,Syndig1,Tpbg,Nrxn3,Clstn2,Sez6l,Unc13b,Ank3,Asic2,Cacnb4,Cux2,Etv5,Malat1,Nedd4,Nrg1,Pdgfb,Pin1,Pten,Sdk2,Adnp,Camk1,Cdh1,Ctnnd2,Dlg4,F2r,Farp1,Thbs2
GO:0060900	embryonic camera-type eye formation	174	Aldh1a3,Sox11,Twist1
GO:0030033	microvillus assembly	207	Podxl,Prl2c2,Fscn1,Rap2a
GO:0051965	positive regulation of synapse assembly	218	Adgrb1,Cbln1,Lrtm2,Syndig1,Tpbg,Nrxn3,Clstn2,Asic2,Cux2,Adnp,Thbs2
GO:0003357	noradrenergic neuron differentiation	228	Ascl1,Insm1,Sox11,Sox4
GO:0014014	negative regulation of gliogenesis	234	Hmga2,Mycn,Dlx2,Nkx6-2,Dab1,Id2,Nf1,Sox11,Bmp4,Tert
GO:0010001	glial cell differentiation	238	Hmga2,Mycn,Nrg1,Olig2,Olig1,Abl1,Abl2,Ascl1,Dlx2,Mapk1,Nab2,Nkx6-2,Ager,Dab1,Id2,Il6st,Miat,Nf1,Pdgfb,Phgdh,Pten,Sox11,Sox4,Sox6,Tenm4,Trpc4,Arhgef10,Aspa,Bmp4,Exoc4,Ncmap,Pparg,Tlr2
GO:0051101	regulation of DNA binding	267	Ankrd33,Fbxw7,Id1,Id2,Msx2,Runx1t1,Zfp462,Zfp90,Ager,E2f1,Hspa4,Mad2l2,Nme1,Park2,Sirt1,Sox11,Twist1
GO:0044091	membrane biogenesis	274	Ptprd,Pten,Ank3,Exoc4
GO:0048709	oligodendrocyte differentiation	310	Olig2,Olig1,Ascl1,Dlx2,Nkx6-2,Id2,Nf1,Nrg1,Pten,Sox6,Tenm4,Trpc4,Aspa,Bmp4,Exoc4,Pparg,Tlr2
GO:0048593	camera-type eye morphogenesis	328	Epha2,Sdk2,Aldh1a3,Bak1,Bax,Col8a1,Fjx1,Aqp1,Aqp5,Bmp4,Dscam,Foxf2,Foxn4,Hcn1,Megf11,Mfn2,Nf1,Sox11,Tdrd7,Tenm3,Twist1
GO:2000793	cell proliferation involved in heart valve development	348	Smad4,Twist1
GO:0061029	eyelid development in camera-type eye	349	Grhl3,Map3k1,Sox11,Twist1
GO:0043392	negative regulation of DNA binding	377	Ankrd33,Fbxw7,Id1,Id2,Msx2,Zfp462,Zfp90,E2f1,Hspa4,Mad2l2,Sirt1,Sox11
GO:0050773	regulation of dendrite development	384	Cit,Cpeb3,Palm,Ptprd,Sdk1,Cux2,Fstl4,Nedd4,Nrg1,Pten,Camk1,Cyth2,Elavl4,Fbxo31,Id1,Kalrn,Numbl,Plk2,Rap2a,Rapgef4,Sema4d,Sipa1l1,Syne1
GO:0050768	negative regulation of neurogenesis	421	Sema4a,Sema4c,Sema4d,Sema6c,Cit,Ctdsp1,Draxin,Gnrh1,H2-D1,Hmga2,Mycn,Nepro,Nrg1,Nrg3,Olig2,Rap1gap2,Syngap1,Dab1,Dlx2,Nkx6-2,Pbx1,Ryk,Arhgef2,Fstl4,Id2,Lmx1a,Nf1,Pten,Sorl1,Sox11,Ascl1,Bmp4,Cyth2,Dab2,Id1,Katna1,Lrp1,Plk2,Rbpj,Sox21,Spock1,Tert,Trpv4
GO:0051961	negative regulation of nervous system development	428	Sema4a,Sema4c,Sema4d,Sema6c,Cit,Ctdsp1,Draxin,Gnrh1,H2-D1,Hmga2,Mycn,Nepro,Nrg1,Nrg3,Olig2,Rap1gap2,Syngap1,Dab1,Dlx2,Nkx6-2,Pbx1,Ryk,Arhgef2,Eif2ak3,Fstl4,Id2,Lmx1a,Nf1,Pten,Sorl1,Sox11,Sufu,Ascl1,Bmp4,Cyth2,Dab2,Id1,Katna1,Lrp1,Plk2,Rbpj,Sox21,Spock1,Tert,Tnf,Trpv4
GO:0048485	sympathetic nervous system development	441	Ascl1,Fzd3,Gdnf,Insm1,Nf1,Sox11,Sox4
GO:0046320	regulation of fatty acid oxidation	447	C1qtnf2,Acacb,Acadvl,Pdk4,Abcd2,Fabp1,Irs1,Pparg,Twist1

GO:0030534	adult behavior	456	Cartpt,Sdk1,Cacna1c,Npc1,Sez6l,a,Abhd12,Adra1b,Cacnb4,Cdh23,Cln8,Dab1,Dbh,Gpr39,Grin2d,Id2,Klhl1,Mafg,Ntf5,Otog,Park2,Pten,Scn8a,Slc1a2,Sptbn4,Tmod1,Tshr,Crhbp,Cxcl12
GO:0045578	negative regulation of B cell differentiation	505	Hmgb3,Id2
GO:0090344	negative regulation of cell aging	529	Abl1,Pten,March5,Sirt1,Tert,Twist1
GO:0048483	autonomic nervous system development	535	Gdnf,Ascl1,Fzd3,Gbx2,Insm1,Nav2,Nf1,Sox11,Sox4
GO:0003254	regulation of membrane depolarization	564	Smad7,Gclc,Gclm,Scn3b,Ank3,Hcn1,Mgea5,Ptpn3,Src,Trpm4
GO:0044272	sulfur compound biosynthetic process	568	Papss2,Acss2,Chst11,Chst3,Gclc,Gclm,Hs6st3,Extl3,Ggt7,Chsy1,Acacb,Eif2ak3,Hs2st1,Mthfr,Pdk1,Pdk4,Slc35d1,Vangl2,Chst12,Chst14,Gss,Mat1a,Mgst2
GO:0044827	modulation by host of viral genome replication	572	Ceacam1,Park2,Ythdc2
GO:0001964	startle response	582	Npas3,Csmd1,Grin2d,Grin3a,Nrg1,Park2,Pten
GO:0032000	positive regulation of fatty acid beta-oxidation	592	Abcd2,Fabp1,Irs1,Twist1
GO:0032434	regulation of proteasomal ubiquitin-dependent protein catabolic process	602	Anapc11,Rnf144a,Smarcc1,Usp44,Bub1b,Aurka,Gclc,Herpud1,Park2,Pten,Apc,Csnk1a1,Csnk2a2,Dab2,Fhit,Mad2l2,Nkd2,Plk1,Plk2,Prickle1,Smad7,Ubqln1,Ubqln2,Ubqln4,Vcp
GO:0090596	sensory organ morphogenesis	607	Epha2,Sdk2,Aldh1a3,Bak1,Bax,Cdh23,Col8a1,Cthrc1,Dvl2,Fjx1,Fzd3,Fzd6,Grhl3,Mapk1,Vangl2,Aqp1,Aqp5,Bhlhe23,Bmp4,Dscam,Fgfr1,Foxf2,Foxn4,Gbx2,Gli2,Hcn1,Itga8,Megf11,Mfn2,Miat,Myo7a,Nf1,Nr2e3,Pdgfb,Ptprq,Rac1,Ror2,Rpl38,Sox11,Tdrd7,Tshr,Tshz1,Ush1c,Wdr19,Tenm3,Twist1
GO:0051963	regulation of synapse assembly	649	Adgrb1,Cbln1,Lrtm2,Syndig1,Tpbg,Nrxn3,Clstn2,Asic2,Cux2,Adnp,Thbs2
GO:0007569	cell aging	654	1500015O10Rik,Prelp,Zmiz1,Abl1,Id2,Pten,Atr,Lmna,March5,Romo1,Sirt1,Tert,Twist1,Wnt16,Zfp217
GO:0048541	Peyer's patch development	662	Id2,Tcf3,Ada,Cacnb4,Ceacam1,Foxl1
GO:0048592	eye morphogenesis	664	Epha2,Sdk2,Aldh1a3,Bak1,Bax,Col8a1,Fjx1,Aqp1,Aqp5,Bhlhe23,Bmp4,Dscam,Foxf2,Foxn4,Hcn1,Megf11,Mfn2,Miat,Nf1,Nr2e3,Pdgfb,Sox11,Tdrd7,Tenm3,Twist1
GO:0048537	mucosal-associated lymphoid tissue development	673	Id2,Tcf3,Ada,Cacnb4,Ceacam1,Foxl1
GO:0042063	gliogenesis	674	Hmga2,Mycn,Nrg1,Olig2,Olig1,Abl1,Abl2,Ascl1,Dlx2,Mapk1,Nab2,Nkx6-2,Ager,Dab1,Hexb,Id2,Il6st,Miat,Nf1,Pdgfb,Phgdh,Pten,Sox11,Sox4,Sox6,Tenm4,Trpc4,Arhgef10,Aspa,Bmp4,Creb1,Etv5,Exoc4,Lta,Ncmap,Pparg,Synj1,Tert,Tgfb2,Tlr2
GO:0046321	positive regulation of fatty acid oxidation	678	C1qtnf2,Abcd2,Fabp1,Irs1,Pparg,Twist1
GO:0001838	embryonic epithelial tube formation	678	Bmp4,Grem1,Cthrc1,Dvl2,Enah,Fzd3,Fzd6,Grhl3,Sall4,Sfrp2,Sfrp5,Vangl2,Gdf7,Gdnf,Grhl2,Hs2st1,Lmo4,Nup50,Rab23,Sema4c,Sox11,Sox4,Sufu,Tead2,Twist1,Prickle1
GO:0072175	epithelial tube formation	681	Bmp4,Grem1,Cthrc1,Dvl2,Enah,Fzd3,Fzd6,Grhl3,Sall4,Sfrp2,Sfrp5,Vangl2,Gdf7,Gdnf,Grhl2,Hs2st1,Lmo4,Nup50,Rab23,Sema4c,Sox11,Sox4,Sufu,Tead2,Twist1,Prickle1
GO:2001223	negative regulation of neuron migration	685	Gnrh1,Nrg1,Nrg3
GO:0090398	cellular senescence	686	1500015O10Rik,Abl1,Id2,Sirt1,Tert,Twist1,Wnt16
GO:0001764	neuron migration	692	Cdh1,Gnrh1,Nrg1,Nrg3,Ascl1,Dab1,Fyn,Ywhae,Barhl1,Barhl2,Bax,Celsr2,Elp3,Fzd3,Katna1,Neurog2,Pex7,Twist1,Cxcl12,Fbxo31,Hsp90aa1
GO:0032528	microvillus organization	707	Podxl,Prl2c2,Vil1,Fscn1,Rap2a
GO:0032436	positive regulation of proteasomal ubiquitin-dependent protein catabolic process	712	Anapc11,Rnf144a,Aurka,Gclc,Park2,Pten,Csnk1a1,Dab2,Herpud1,Nkd2,Plk1,Plk2,Prickle1,Smad7,Ubqln1,Ubqln2,Vcp

Supplementary Table S5. Top 50 Biological Processes Gene Ontology terms associated with shared mouse F0 and F1 methylation clusters. Table reports terms (p-value<0.01) selected after filtering for a number of associated genes >1 and < 50 and ranked according to geometrical mean of ranks in 100 randomizations.

GO ID	Term	Rank product	Associated genes
GO:0030336	negative regulation of cell migration	44	Erdr1,Marveld3,Nrg3,Rap2a,Pdgfb
GO:2000146	negative regulation of cell motility	63	Erdr1,Marveld3,Nrg3,Rap2a,Pdgfb
GO:0031954	positive regulation of protein autophosphorylation	69	Pdgfb,Rap2a
GO:0050919	negative chemotaxis	156	Nrg3,Slit3
GO:0051271	negative regulation of cellular component movement	156	Erdr1,Marveld3,Nrg3,Rap2a,Pdgfb
GO:0040013	negative regulation of locomotion	249	Erdr1,Marveld3,Nrg3,Rap2a,Pdgfb
GO:0048541	Peyer's patch development	281	Tcf3,Cacnb4,Ceacam1
GO:0006910	phagocytosis, recognition	283	Ighv1-83,Ighv1-84
GO:0048537	mucosal-associated lymphoid tissue development	298	Tcf3,Cacnb4,Ceacam1
GO:0050773	regulation of dendrite development	340	Sdk1,Kalrn,Rap2a
GO:0006909	phagocytosis	378	Ighv1-83,Ighv1-84,Mbl1,Sftpa1,Sftpd
GO:0032703	negative regulation of interleukin-2 production	394	Ceacam1,Sftpd
GO:0050767	regulation of neurogenesis	493	Slit3,Nrg3,Sdk1,Syngap1,Tcf3,Tcf4,Fzd3,Enc1,Kalrn,Nkx2-5,Rap2a,Synj1,Tert,Trpv4
GO:0040012	regulation of locomotion	543	Erdr1,Marveld3,Nrg3,Rap2a,Pdgfb,Tff2,Elp3,Lrrc16a,Mir218-2,Trpv4,Tshr,F3,Tert
GO:0035264	multicellular organism growth	553	Heg1,Slc1a2,Stc2,Tshr
GO:0046328	regulation of JNK cascade	555	Ceacam1,Sfrp5,Trpv4,Marveld3,Rap2a
GO:0051960	regulation of nervous system development	574	Slit3,Nrg3,Sdk1,Syngap1,Tcf3,Tcf4,Sfrp5,Eif2ak3,Fzd3,Enc1,Kalrn,Nkx2-5,Rap2a,Synj1,Tert,Trpv4
GO:0030334	regulation of cell migration	579	Erdr1,Marveld3,Nrg3,Rap2a,Pdgfb,Tff2,Elp3,Lrrc16a,Mir218-2,Trpv4,F3,Tert
GO:0007254	JNK cascade	605	Ceacam1,Sfrp5,Trpv4,Marveld3,Rap2a
GO:0060284	regulation of cell development	628	Slit3,Nrg3,Sdk1,Syngap1,Tcf3,Tcf4,Fzd3,Lrrc16a,Enc1,Kalrn,Nkx2-5,Rap2a,S1pr3,Synj1,Tert,Trpv4
GO:2000145	regulation of cell motility	630	Erdr1,Marveld3,Nrg3,Rap2a,Pdgfb,Tff2,Elp3,Lrrc16a,Mir218-2,Trpv4,F3,Tert
GO:0010629	negative regulation of gene expression	635	Dydc1,Hist1h2aa,Hist1h2al,Nacc2,Eif2ak3,Glis3,Mbd3,Mir218-2,Nkx2-5,Tcf4,Sall4,Slit3,Acacb,Tax1bp1,Trpv4,Jazf1,Bak1,Cbx6,Cbx7,Enc1,Hcfc2,Kctd1,Mvk,Pdgfb,Rwdd3,Sfrp5,Stc2,Tcf3,Tert,Zfp148,Zfp217,Mex3d
GO:0032872	regulation of stress-activated MAPK cascade	653	Ceacam1,Sfrp5,Trpv4,Marveld3,Rap2a
GO:0070302	regulation of stress-activated protein kinase signaling cascade	656	Ceacam1,Sfrp5,Trpv4,Marveld3,Rap2a
GO:0035385	Roundabout signaling pathway	658	Slit3,Mir218-2
GO:0001764	neuron migration	676	Nrg3,Elp3,Fzd3
GO:0010842	retina layer formation	704	Fjx1,Foxn4
GO:0048468	cell development	706	Slit3,Nrg3,Sdk1,Syngap1,Tcf3,Tcf4,Taf4b,Bak1,Heg1,Lrrk1,Nkx2-5,Sycp3,Ank3,Arhgef28,Cacnb4,Chst11,Chsy1,Crtac1,Eif2ak3,Fzd3,Hexb,Hydin,Lrrc16a,Miat,Myo18b,Pdgfb,Ropn1,Rsph1,Sohlh1,Trip13,Tshr,Camsap1,Enc1,Kalrn,Rap2a,S1pr3,Synj1,Tert,Tjp1,Trpv4,Zfyve27
GO:0051270	regulation of cellular component movement	712	Erdr1,Marveld3,Nrg3,Rap2a,Pdgfb,Tff2,Elp3,Lrrc16a,Mir218-2,Trpv4,F3,Tert
GO:0046415	urate metabolic process	726	Lrrc16a,Slc16a9
GO:1902805	positive regulation of synaptic vesicle transport	743	Unc13b,Synj1

GO:0048172	regulation of short-term neuronal synaptic plasticity	751	Syngr1,Unc13b
GO:0007399	nervous system development	758	Slit3,Zfp217,Dok5,Nrg3,Sdk1,Syngap1,Tcf3,Tcf4,Elp3,Enc1,Foxn4,Fzd3,Shc3,Hexb,Sall4,Sez6l,Sfrp5,Unc13b,Aldh1a3,Ank3,Arhgef28,Cables1,Crtac1,Eif2ak3,Hydin,Lemd2,Miat,Pdgfb,Slc1a2,Tshr,Camsap1,Kalrn,Nkx2-5,Rap2a,Synj1,Tert,Trpv4,Zfyve27
GO:0009794	regulation of mitotic cell cycle, embryonic	774	Pdgfb,Fzd3
GO:0045448	mitotic cell cycle, embryonic	790	Pdgfb,Fzd3
GO:0036342	post-anal tail morphogenesis	805	Sfrp5,Chst11,Fzd3
GO:0051403	stress-activated MAPK cascade	827	Ceacam1,Sfrp5,Trpv4,Marveld3,Rap2a
GO:0016358	dendrite development	832	Sdk1,Syngap1,Kalrn,Rap2a
GO:0032879	regulation of localization	834	Cacnb4,Abcg1,Erdr1,Marveld3,Mrln,Nrg3,Rap2a,Bak1,Mbl1,Pdgfb,Tff2,Acacb,Ank3,Ceacam1,Eif2ak3,Elp3,Lrrc16a,Mir218-2,Nkx2-5,Slc1a2,Stc2,Tert,Trpv4,Uqcc2,F3,Hps4,Mis18a,Mybpc1,Nkd2,Sfrp5,Sftpa1,Sftpd,Synj1,Unc13b
GO:0006958	complement activation, classical pathway	836	Ighv1-83,Ighv1-84,Mbl1
GO:0050851	antigen receptor-mediated signaling pathway	836	Ighv1-83,Ighv1-84,Ubash3a,Cacnb4
GO:2000505	regulation of energy homeostasis	838	Ceacam2,Trpv4
GO:0030534	adult behavior	842	Sdk1,Sez6l,Cacnb4,Slc1a2,Tshr
GO:0040014	regulation of multicellular organism growth	875	Stc2,Tshr
GO:0002429	immune response-activating cell surface receptor signaling pathway	884	Ighv1-83,Ighv1-84,Ubash3a,Cacnb4
GO:0048699	generation of neurons	903	Slit3,Nrg3,Sdk1,Syngap1,Tcf3,Tcf4,Foxn4,Ank3,Arhgef28,Crtac1,Elp3,Fzd3,Miat,Pdgfb,Tshr,Camsap1,Enc1,Kalrn,Nkx2-5,Rap2a,Synj1,Tert,Trpv4,Zfyve27
GO:0007143	female meiotic division	905	Spin1,Sycp3,Trip13
GO:0002768	immune response-regulating cell surface receptor signaling pathway	909	Ighv1-83,Ighv1-84,Ubash3a,Cacnb4
GO:0038084	vascular endothelial growth factor signaling pathway	911	Tcf4,Pdgfb
GO:0016082	synaptic vesicle priming	919	Synj1,Unc13b

Supplementary Table S6.
DMRs (Wilcoxon p-value<0.05) resulting from within-pair comparisons of MZ discordant twins (responder versus non-responder twin) as compared to data from MZ twins pairs' concordant for absence of CO_2 hypersensitivity.

**chr**	**start**	**end**	**logFC**	**p-value**	**Wilcoxon p-value**	**gene ID**	**insideFeature**	gene name
chr1	10788536	10788989	-0.518717526	0.000616503	0.046955985	ENSG00000130940	inside	CASZ1
chr1	17113039	17114068	-0,57597551	0,000770614	0,001378034	ENSG00000058453	inside	CROCC
chr1	29361480	29361780	0,662154438	0,000274193	0,003887289	ENSG00000159023	inside	EPB41
chr1	30378654	30378977	0,858040375	0,000499178	0,038502674	ENSG00000270927	upstream	RP11-179G8,1
chr1	30572610	30572890	0,810542186	0,000764447	0,009378856	ENSG00000233372	upstream	RP5-893G23,1
chr1	45363389	45363656	0,991634466	3,44E-05	0,00281777	ENSG00000070785	inside	EIF2B3
chr1	45818442	45818861	0,659697332	0,000181344	0,000390786	ENSG00000070759	inside	TESK2
chr1	46727654	46728037	0,881773551	0,00058551	0,031262855	ENSG00000085999	inside	RAD54L
chr1	62037057	62037425	-1,146139899	0,000741193	0,025154258	ENSG00000264551	upstream	AC099791,1
chr1	64803758	64804098	1,17377593	0,000695469	0,019991773	ENSG00000238653	downstream	RNU7-62P
chr1	67588625	67588977	0,854968226	0,000461233	0,015734266	ENSG00000203963	inside	C1orf141
chr1	69463308	69463655	0,804229917	0,000226284	0,012217195	ENSG00000223883	upstream	RP11-424D14,1
chr1	85330224	85330535	0,77942129	0,000791451	0,005306458	ENSG00000171517	inside	LPAR3
chr1	95162701	95163065	0,714641638	0,000335925	0,009378856	ENSG00000224081	inside	LINC01057
chr1	103083245	103083577	0,71896727	5,60E-05	0,046955985	ENSG00000233359	upstream	RP11-202K23,1
chr1	109751806	109752127	0,840373863	0,00073865	0,038502674	ENSG00000238310	upstream	RNU7-122P
chr1	115042499	115042898	1,129788571	1,91E-05	0,038502674	ENSG00000197323	inside	TRIM33
chr1	193268820	193269235	-0,907167229	0,000681925	0,038502674	ENSG00000232077	upstream	LINC01031
chr1	201387427	201387697	0,608471589	0,000683439	0,046955985	ENSG00000159173	inside	TNNI1
chr1	213530944	213531322	0,640298896	0,000323874	0,038502674	ENSG00000228646	downstream	RPL31P13
chr1	229764518	229764778	0,933657042	0,000996578	0,046955985	ENSG00000135763	inside	URB2
chr1	244846446	244846979	0,632904525	0,000370787	0,012217195	ENSG00000121644	inside	DESI2
chr10	2018816	2019099	0,969158649	6,74E-05	0,019991773	ENSG00000234962	downstream	LINC00700
chr10	24327547	24327886	-0,700179344	0,000336214	0,038502674	ENSG00000120549	inside	KIAA1217
chr10	35815680	35816034	0,534581028	0,000511753	0,031262855	ENSG00000108100	inside	CCNY
chr10	53661882	53662192	0,996097567	0,000619763	0,009378856	ENSG00000185532	inside	PRKG1
chr10	65002940	65003439	-0,726189191	0,000271343	0,046955985	ENSG00000171988	inside	JMJD1C
chr10	69864504	69864895	0,938149893	0,000162731	0,005306458	ENSG00000138347	upstream	MYPN
chr10	78487989	78488397	0,72090977	0,000134755	0,015734266	ENSG00000252888	downstream	SNORA31
chr10	84048452	84048801	1,161370312	0,000352704	0,025154258	ENSG00000185737	inside	NRG3
chr10	86772823	86773371	-0,572626423	0,000500481	0,038502674	ENSG00000238469	downstream	AC091487,1
chr10	102302004	102302494	-0,498847333	0,000353748	0,015734266	ENSG00000166136	upstream	NDUFB8
chr10	113100839	113101197	1,178610467	0,00053285	0,007095845	ENSG00000227851	downstream	RP11-381K7,1
chr10	113584178	113584580	1,083749155	0,000811155	0,000925545	ENSG00000230809	upstream	RP11-309P22,1
chr10	118791631	118791926	0,987469561	0,000628315	0,000925545	ENSG00000187164	inside	KIAA1598
chr10	120058756	120059160	0,645289286	0,000487811	0,019991773	ENSG00000165669	downstream	FAM204A
chr10	127412168	127413246	0,651426472	0,000815111	0,038502674	ENSG00000224023	upstream	RP11-383C5,4

chr10	130290513	130290947	0,588258016	0,000237346	0,025154258	ENSG00000234640	upstream	RP11-264E18,1
chr11	2691130	2691604	0,989444359	0,000731986	0,031262855	ENSG00000053918	inside	KCNQ1
chr11	41813994	41814404	0,564045587	0,000369375	0,012217195	ENSG00000255171	inside	RP11-375D13,2
chr11	43329525	43329783	-0,954364008	4,84E-05	0,003887289	ENSG00000166181	upstream	API5
chr11	62709357	62709851	-0,738633295	0,000513691	0,015734266	ENSG00000241082	upstream	RN7SL259P
chr11	71441798	71442138	0,887415276	0,000983785	0,046955985	ENSG00000255319	inside	ENPP7P8
chr11	87119358	87119676	0,761587293	0,000785237	0,031262855	ENSG00000255391	downstream	CTD-2028E8,1
chr11	88367914	88368251	0,954193818	0,000120625	0,025154258	ENSG00000168959	inside	GRM5
chr11	104696325	104696646	1,101329326	0,000257424	0,019991773	ENSG00000254569	upstream	RP11-693N9,1
chr11	111688230	111688849	0,488627552	0,000315272	0,003887289	ENSG00000086848	inside	ALG9
chr11	115604881	115605206	0,624159138	0,000305796	0,031262855	ENSG00000256717	inside	AP000797,3
chr11	120184350	120184840	0,651562623	0,000857364	0,046955985	ENSG00000137709	inside	POU2F3
chr11	132027718	132028024	0,93521026	0,000258002	0,003887289	ENSG00000182667	inside	NTM
chr12	17272869	17273272	0,994589796	0,000189522	0,038502674	ENSG00000242991	upstream	RPL7P40
chr12	22791319	22791587	0,687182624	0,000422034	0,038502674	ENSG00000139163	inside	ETNK1
chr12	25175747	25176003	0,895785754	2,24E-05	0,00281777	ENSG00000118308	inside	LRMP
chr12	64882442	64882735	0,767029702	0,000183434	0,031262855	ENSG00000183735	inside	TBK1
chr12	83956310	83956623	-0,726127478	6,53E-05	0,005306458	ENSG00000257124	downstream	RP11-384P14,1
chr12	87512289	87512581	0,992278414	0,000218052	0,015734266	ENSG00000242850	downstream	RPL23AP68
chr12	95013666	95014239	0,495676952	0,000674554	0,019991773	ENSG00000057704	inside	TMCC3
chr12	97027777	97028281	0,656622621	0,000552834	0,001995064	ENSG00000188596	inside	C12orf55
chr12	112278223	112278486	0,575315532	0,000743861	0,00281777	ENSG00000234608	inside	MAPKAPK5-AS1
chr12	125803740	125803996	0,787357132	0,000499067	0,019991773	ENSG00000139364	inside	TMEM132B
chr13	20584325	20584642	0,775151957	6,48E-05	0,031262855	ENSG00000121741	inside	ZMYM2
chr13	20768858	20769212	0,661640376	0,000950505	0,038502674	ENSG00000165474	upstream	GJB2
chr13	21031591	21032130	0,845641861	0,000222713	0,005306458	ENSG00000165475	inside	CRYL1
chr13	24747173	24747429	0,760711807	0,00064699	0,046955985	ENSG00000273167	inside	RP11-307N16,6
chr13	27987711	27988213	0,678027091	0,000172665	0,046955985	ENSG00000122034	upstream	GTF3A
chr13	28251382	28251709	1,071545752	7,10E-05	0,000143974	ENSG00000226703	downstream	NPM1P4
chr13	30184678	30185270	0,725493277	5,00E-05	0,001995064	ENSG00000139514	upstream	SLC7A1
chr13	33777073	33777376	0,967840274	0,000704126	0,019991773	ENSG00000133121	inside	STARD13
chr13	49763404	49763704	0,783250963	0,000696797	0,003887289	ENSG00000102531	inside	FNDC3A
chr13	52540415	52540696	0,763625678	0,000701135	0,00061703	ENSG00000123191	inside	ATP7B
chr13	52638736	52639057	0,705132503	0,000406763	0,031262855	ENSG00000253797	downstream	UTP14C
chr13	73690560	73690902	1,060977216	0,000111202	0,007095845	ENSG00000200267	upstream	RNU6-66P
chr13	74684934	74685204	1,002844996	0,000111675	0,007095845	ENSG00000118922	inside	KLF12
chr13	100020912	100021229	0,705299781	0,000441351	0,003887289	ENSG00000134882	inside	UBAC2
chr13	100765925	100766218	0,765431944	0,000157865	0,025154258	ENSG00000175198	inside	PCCA
chr13	103284136	103284444	0,851129331	0,000405256	0,025154258	ENSG00000134900	inside	TPP2
chr13	109764054	109764318	0,949305396	0,000376792	0,009378856	ENSG00000041515	inside	MYO16
chr14	34174630	34174976	0,943705202	0,000507365	0,015734266	ENSG00000151322	inside	NPAS3
chr14	39060305	39060573	1,072999702	0,000301016	0,019991773	ENSG00000271468	upstream	RP11-14N4,1
chr14	57486247	57486553	0,714791352	0,000116359	0,019991773	ENSG00000248550	inside	OTX2-AS1

chr14	84779896	84780190	1,003469766	0,000971434	0,031262855	ENSG00000259012	downstream	CTD-2320B12,2
chr14	85147562	85147880	0,794065407	0,000318184	0,046955985	ENSG00000251895	upstream	RNU6-976P
chr15	20591865	20592132	0,962697845	0,000158994	0,015734266	ENSG00000265002	downstream	AC026495,1
chr15	44682763	44683031	0,865861243	0,000582238	0,005306458	ENSG00000166734	inside	CASC4
chr15	58923414	58923689	0,929551429	0,000724693	0,046955985	ENSG00000137845	inside	ADAM10
chr15	85288661	85289078	0,805266415	0,000256695	0,038502674	ENSG00000166716	upstream	ZNF592
chr15	91641505	91641773	0,892015762	0,000251373	0,003887289	ENSG00000185518	upstream	SV2B
chr16	9903599	9904119	-0,523131921	0,000475688	0,025154258	ENSG00000260432	upstream	RP11-297M9,2
chr16	17991842	17992275	0,452566239	0,000866095	0,046955985	ENSG00000228071	upstream	RPL7P47
chr16	28816236	28816728	0,719154089	0,000228369	0,046955985	ENSG00000251417	inside	RP11-1348G14,4
chr16	55148558	55148845	0,653497411	0,000440345	0,046955985	ENSG00000260147	downstream	CTD-3032H12,3
chr16	77682580	77682841	0,87608188	0,000435361	0,007095845	ENSG00000261154	downstream	RP11-571O6,1
chr16	85615019	85615307	0,537524345	0,000669761	0,025154258	ENSG00000270124	inside	RP11-118F19,1
chr17	10794565	10794899	0,901189863	0,000493338	0,00281777	ENSG00000244753	upstream	RPL15P21
chr17	22067609	22068053	0,743011595	0,000489065	0,025154258	ENSG00000264930	upstream	RP11-846F4,10
chr17	32305217	32305485	0,908887882	0,000226582	0,012217195	ENSG00000108684	inside	ASIC2
chr17	37277955	37278235	0,736179657	0,000163255	0,025154258	ENSG00000161381	inside	PLXDC1
chr17	38630195	38630455	-0,495016136	0,000845929	0,046955985	ENSG00000131746	downstream	TNS4
chr17	58945501	58945760	1,147887968	0,000823854	0,012217195	ENSG00000141376	inside	BCAS3
chr17	63683305	63683692	0,652814802	0,000142657	0,005306458	ENSG00000154240	inside	CEP112
chr18	9422023	9422596	-0,594106371	0,000416168	0,031262855	ENSG00000273335	downstream	RP11-61L19,2
chr18	10505146	10505605	-0,933896133	0,000126553	0,007095845	ENSG00000134265	upstream	NAPG
chr18	10589647	10590311	0,633476725	0,000170031	0,038502674	ENSG00000265728	upstream	RP11-883A18,3
chr18	29164430	29164695	1,397165171	8,98E-05	0,012217195	ENSG00000118271	upstream	TTR
chr18	35450350	35450681	0,839668416	0,000724203	0,046955985	ENSG00000243516	downstream	RP11-19F9,1
chr18	49941867	49942183	0,780760118	0,000113613	0,025154258	ENSG00000187323	inside	DCC
chr18	51746207	51746569	0,686654335	0,000370065	0,046955985	ENSG00000134046	inside	MBD2
chr19	22027447	22027741	0,844813404	3,85E-05	0,046955985	ENSG00000198521	inside	ZNF43
chr19	29034594	29034900	0,795167788	0,000160351	0,025154258	ENSG00000267243	inside	AC005307,3
chr19	34278837	34279134	0,900201111	4,07E-05	0,007095845	ENSG00000153885	upstream	KCTD15
chr19	34580288	34580761	0,546015707	0,000828786	0,046955985	ENSG00000186008	downstream	RPS4XP21
chr19	34784596	34784883	0,571393621	0,000411914	0,003887289	ENSG00000166398	inside	KIAA0355
chr19	56770570	56771013	-0,706799046	5,31E-05	0,00281777	ENSG00000131848	inside	ZSCAN5A
chr2	15639211	15639479	1,036756858	6,61E-05	0,031262855	ENSG00000151779	inside	NBAS
chr2	15933918	15934397	0,515080879	0,00058657	0,031262855	ENSG00000237326	downstream	AC113608,1
chr2	16474510	16475113	0,796820081	0,000329684	0,031262855	ENSG00000232444	downstream	AC010745,4
chr2	20699899	20700181	-0,750856605	0,000645582	0,019991773	ENSG00000227047	inside	AC012065,4
chr2	32047610	32047878	0,963477503	0,000300742	0,025154258	ENSG00000237007	overlapEnd	KRT18P52
chr2	35851438	35851703	1,228037295	0,000832137	0,012217195	ENSG00000234587	downstream	MRPL50P1
chr2	49076351	49076610	0,82360853	0,000907175	0,046955985	ENSG00000214602	downstream	CTBP2P5

chr2	49736355	49736632	0,879735667	0,000606453	0,038502674	ENSG00000206915	upstream	RNU6-439P
chr2	55729381	55730147	-0,555559652	0,000634542	0,038502674	ENSG00000239189	downstream	RNU6-634P
chr2	90253645	90253977	1,061644521	0,000287528	0,005306458	ENSG00000242580	downstream	IGKV1D-43
chr2	103400266	103400525	1,098204047	0,000722146	0,009378856	ENSG00000170417	inside	TMEM182
chr2	109192808	109193076	0,88222789	0,000514274	0,031262855	ENSG00000169756	inside	LIMS1
chr2	111513669	111514125	0,681118633	0,000906039	0,031262855	ENSG00000153093	inside	ACOXL
chr2	115934772	115935054	0,96122026	5,33E-05	0,019991773	ENSG00000175497	inside	DPP10
chr2	147179571	147179915	0,724073689	0,000301128	0,025154258	ENSG00000227134	upstream	AC093084,1
chr2	160145165	160145588	0,561895124	0,000739979	0,015734266	ENSG00000196151	upstream	WDSUB1
chr2	160210921	160211217	1,011909115	0,000223742	0,025154258	ENSG00000123636	inside	BAZ2B
chr2	161166995	161167409	0,878930027	0,000260369	0,046955985	ENSG00000115221	upstream	ITGB6
chr2	168859147	168859893	-0,520450516	0,000513045	0,015734266	ENSG00000235335	upstream	AC016723,4
chr2	179625014	179625359	0,882274553	0,000340255	0,009378856	ENSG00000237298	inside	TTN-AS1
chr2	193103022	193103376	1,038212322	0,000254505	0,025154258	ENSG00000144339	upstream	TMEFF2
chr2	207847050	207847342	0,849105169	0,000560413	0,019991773	ENSG00000144410	downstream	CPO
chr2	209883061	209883396	0,988530549	2,68E-05	0,005306458	ENSG00000235864	downstream	HSPA8P6
chr2	220220559	220220846	0,978023192	0,000477207	0,019991773	ENSG00000232789	downstream	AC053503,2
chr2	234976015	234976608	-0,533717892	0,000776114	0,038502674	ENSG00000072080	inside	SPP2
chr20	11494518	11494977	-0,851365439	0,000953845	0,015734266	ENSG00000271397	downstream	RP5-1128N12,2
chr20	13134656	13135294	0,599059599	0,000842051	0,038502674	ENSG00000172296	inside	SPTLC3
chr20	20110555	20110858	0,758636207	0,000236287	0,015734266	ENSG00000089101	inside	C20orf26
chr20	39671207	39671475	0,962103188	9,22E-05	0,019991773	ENSG00000198900	inside	TOP1
chr20	53711171	53711560	0,739834764	0,000106987	0,046955985	ENSG00000185834	downstream	RPL12P4
chr20	55544099	55544525	0,811138493	0,000272161	0,025154258	ENSG00000251772	upstream	AL117380,2
chr21	40093449	40093705	0,994822516	0,000225177	0,00281777	ENSG00000223806	downstream	LINC00114
chr22	20015204	20015460	-0,580629077	9,54E-05	0,031262855	ENSG00000183597	inside	TANGO2
chr22	26025243	26025511	0,95369337	0,000687296	0,019991773	ENSG00000100077	inside	ADRBK2
chr3	4000946	4001240	0,860238039	0,000967986	0,025154258	ENSG00000144455	inside	SUMF1
chr3	4734525	4734813	1,204440232	0,000724864	0,019991773	ENSG00000150995	inside	ITPR1
chr3	36408953	36409464	-0,499746524	0,00058381	0,015734266	ENSG00000144681	upstream	STAC
chr3	49373844	49374250	0,548241492	0,000632404	0,003887289	ENSG00000114316	inside	USP4
chr3	50735733	50736082	-0,966784888	0,000143963	0,025154258	ENSG00000088538	inside	DOCK3
chr3	70895177	70895441	-1,242736827	0,000121016	0,046955985	ENSG00000221382	downstream	AC096971,1
chr3	77477310	77477945	-0,676784519	3,70E-05	0,001378034	ENSG00000185008	inside	ROBO2
chr3	122348072	122348382	1,167916035	0,00013916	0,025154258	ENSG00000173200	inside	PARP15
chr3	180730020	180730393	0,971473938	4,30E-05	0,019991773	ENSG00000205981	upstream	DNAJC19
chr4	7623085	7623353	1,148613151	0,000678011	0,00281777	ENSG00000184985	inside	SORCS2
chr4	12714071	12714424	0,846002962	0,000641541	0,019991773	ENSG00000249780	upstream	RP11-352E6,2
chr4	17276468	17276903	-0,710648747	0,000927075	0,015734266	ENSG00000206780	downstream	SNORA75
chr4	20311271	20311903	-0,657569648	0,00012155	0,007095845	ENSG00000145147	inside	SLIT2
chr4	20513573	20513915	0,719505228	0,000818453	0,012217195	ENSG00000145147	inside	SLIT2
chr4	36786050	36786326	0,915791665	5,75E-05	0,009378856	ENSG00000248215	upstream	RP11-722M1,1

chr4	57048175	57048452	0,934894582	0,000156668	0,019991773	ENSG00000109265	inside	KIAA1211
chr4	65802017	65802276	0,91213691	2,12E-05	0,007095845	ENSG00000250125	inside	RP11-707A18,1
chr4	88181917	88182371	0,786009154	0,000490667	0,038502674	ENSG00000250572	downstream	RP11-529H2,1
chr4	109684789	109685332	-0,550324633	0,000169975	0,025154258	ENSG00000164089	upstream	ETNPPL
chr4	113535517	113536126	-0,506548785	0,000965221	0,009378856	ENSG00000138658	inside	C4orf21
chr4	162382183	162382718	-0,585552927	0,000925345	0,038502674	ENSG00000249568	downstream	RP11-234O6,2
chr4	163321496	163321764	1,372752766	0,000371478	0,000390786	ENSG00000251253	downstream	MTHFD2P4
chr4	184755519	184755908	0,736817149	0,000719953	0,038502674	ENSG00000173320	upstream	STOX2
chr5	1448832	1449107	1,097970227	0,000696789	0,005306458	ENSG00000142319	upstream	SLC6A3
chr5	6746211	6746552	0,553974764	0,000833842	0,038502674	ENSG00000112941	inside	PAPD7
chr5	28668392	28668794	0,781641578	0,000520293	0,025154258	ENSG00000252867	downstream	RNU6-909P
chr5	34658581	34659007	0,660960961	0,000429854	0,007095845	ENSG00000039560	inside	RAI14
chr5	35190304	35190665	1,193917762	0,000279643	0,005306458	ENSG00000113494	inside	PRLR
chr5	42130522	42130983	-0,694900881	0,00042961	0,009378856	ENSG00000260786	upstream	RP11-112L7,1
chr5	107433244	107433531	0,945679905	3,64E-05	0,000390786	ENSG00000145743	inside	FBXL17
chr5	154680618	154680964	0,873501192	0,00050225	0,003887289	ENSG00000271477	upstream	CTC-447K7,1
chr5	155955992	155956377	0,572824719	0,000516514	0,038502674	ENSG00000170624	inside	SGCD
chr5	157259885	157260337	0,689629501	0,000841774	0,025154258	ENSG00000113282	inside	CLINT1
chr5	157495305	157495607	0,86730388	0,000172297	0,00281777	ENSG00000253422	downstream	CTB-47B8,4
chr5	166689174	166689464	1,091429224	0,000340897	0,025154258	ENSG00000145934	upstream	TENM2
chr6	11815101	11815489	0,646777744	0,000528793	0,009378856	ENSG00000234427	downstream	RP3-413H6,2
chr6	25102839	25103203	0,947901514	0,000428573	0,019991773	ENSG00000168405	inside	CMAHP
chr6	25435384	25435779	0,639605253	0,000293917	0,019991773	ENSG00000079691	inside	LRRC16A
chr6	66250463	66250912	-0,586962152	0,000711381	0,019991773	ENSG00000188107	inside	EYS
chr6	105105451	105105833	-0,806958198	0,000742199	0,031262855	ENSG00000218173	upstream	RP11-427E4,1
chr6	109121897	109122153	0,990185558	0,000317456	0,031262855	ENSG00000219565	upstream	ZNF259P1
chr6	115883500	115883834	1,021039756	0,000110987	0,046955985	ENSG00000228777	downstream	RP11-282C5,1
chr6	142067894	142068551	-0,531921373	0,00070052	0,031262855	ENSG00000218351	upstream	RPS3AP23
chr6	158221359	158221682	1,280804374	6,81E-05	0,015734266	ENSG00000130340	upstream	SNX9
chr6	170233615	170233969	1,052733518	0,000714593	0,009378856	ENSG00000236173	upstream	RP1-182D15,2
chr7	16142167	16142426	0,968729407	0,000897135	0,031262855	ENSG00000273477	upstream	RP11-196O16,1
chr7	16632708	16633091	0,730055154	0,000745664	0,009378856	ENSG00000272361	downstream	GS1-166A23,2
chr7	21864484	21864958	0,530260089	0,000820011	0,019991773	ENSG00000105877	inside	DNAH11
chr7	35525620	35525899	1,133694171	1,66E-05	0,015734266	ENSG00000235464	downstream	AC007652,1
chr7	46010878	46011165	0,748317975	0,000909239	0,019991773	ENSG00000237471	inside	AC073115,6
chr7	54808563	54809041	-0,91899378	7,98E-05	0,009378856	ENSG00000234707	upstream	RP11-745C15,2
chr7	54815369	54815922	-0,532921392	0,000931482	0,031262855	ENSG00000234707	upstream	RP11-745C15,2
chr7	85759587	85759854	0,995018064	0,000157562	0,019991773	ENSG00000236610	downstream	SOCS5P1
chr7	107716187	107716503	0,85193457	0,000873983	0,031262855	ENSG00000091128	inside	LAMB4
chr7	137000819	137001078	1,01435025	0,00022795	0,031262855	ENSG00000105894	inside	PTN

chr7	152573854	152574175	0,668387847	0,000292855	0,009378856	ENSG00000133627	downstream	ACTR3B
chr7	154601318	154601585	0,682555365	0,000668197	0,025154258	ENSG00000130226	inside	DPP6
chr8	7733663	7733979	0,806802849	0,000339717	0,009378856	ENSG00000176797	upstream	DEFB103A
chr8	15011171	15011686	-0,644723062	0,000216268	0,012217195	ENSG00000185053	inside	SGCZ
chr8	18960224	18960616	-0,657716193	0,00034053	0,019991773	ENSG00000253557	inside	RP11-1080G15,1
chr8	23454529	23454785	2,1498655	0,000595984	0,025154258	ENSG00000252067	downstream	RNU4-71P
chr8	50847774	50848046	1,13211192	4,39E-05	0,046955985	ENSG00000147481	inside	SNTG1
chr8	102682840	102683189	0,812473279	0,000100018	0,031262855	ENSG00000253629	upstream	KB-1107E3,1
chr8	118846568	118847058	-0,86329343	0,000506124	0,005306458	ENSG00000182197	inside	EXT1
chr8	125265808	125266091	1,047995338	0,000108165	0,015734266	ENSG00000214803	upstream	RP11-37N22,1
chr8	137250308	137250567	0,759926672	0,000581734	0,046955985	ENSG00000253248	downstream	RP11-149P24,1
chr9	21890843	21891125	1,302073525	0,000905812	0,005306458	ENSG00000099810	inside	MTAP
chr9	29870117	29870657	0,685241913	0,000931154	0,003887289	ENSG00000230097	upstream	RP11-460C6,1
chr9	36753286	36753840	-0,900657919	0,000163783	0,009378856	ENSG00000266255	downstream	MIR4475
chr9	42171212	42171498	0,811855755	0,0002198	0,046955985	ENSG00000184961	upstream	AL772307,1
chr9	71346794	71347117	0,846272764	0,000774318	0,019991773	ENSG00000107242	inside	PIP5K1B
chr9	94182449	94183208	0,87816236	0,000800948	0,019991773	ENSG00000165030	inside	NFIL3
chr9	113210247	113210685	0,622389724	0,000335529	0,025154258	ENSG00000165124	inside	SVEP1
chr9	130130527	130131193	0,651939462	0,000844581	0,046955985	ENSG00000136895	inside	GARNL3
chr9	135505220	135506112	0,536948091	0,000393019	0,038502674	ENSG00000125485	inside	DDX31
chrX	5661195	5661455	1,261876262	0,000146215	0,000390786	ENSG00000236120	upstream	RP11-733O18,1
chrX	7104325	7104589	1,152977595	0,000334823	0,046955985	ENSG00000101846	upstream	STS
chrX	13123605	13124125	0,516233825	0,000863475	0,025154258	ENSG00000261030	downstream	RP11-791M20,1
chrX	15764640	15765013	0,625323014	0,000867777	0,046955985	ENSG00000169239	inside	CA5B
chrX	33401596	33401866	1,120498921	0,00042314	0,038502674	ENSG00000215310	upstream	RP6-60B16,1
chrX	54484788	54485047	0,573683917	0,000569721	0,038502674	ENSG00000158526	downstream	TSR2
chrX	96232725	96233047	1,52752618	0,000905594	0,046955985	ENSG00000147202	inside	DIAPH2
chrX	99299007	99299263	1,31970065	5,90E-05	0,007095845	ENSG00000252296	upstream	SNORA25
chrX	121830955	121831515	-0,673910253	3,99E-05	0,000390786	ENSG00000212321	upstream	U3
chrX	130972208	130972526	-1,009268264	0,000797768	0,019991773	ENSG00000213468	upstream	RP11-453F18__B,1
chrX	133917336	133917625	0,762174024	0,000140868	0,031262855	ENSG00000156504	inside	FAM122B

Supplementary Table S7. Top 50 GO terms associated with genes annotated to MZ twins methylation clusters (Biological Processes database). Terms (p-value<0.01) have been selected after filtering for a number of associated genes >1 and < 50 and ranked according to geometrical mean of ranks in 100 randomizations.

GO ID	Term	Rank product	Associated genes
GO:0033563	dorsal/ventral axon guidance	32	DCC,SLIT2
GO:0061364	apoptotic process involved in luteolysis	50	ROBO2,SLIT2
GO:0021772	olfactory bulb development	57	SLIT2,EXT1,ROBO2
GO:0021988	olfactory lobe development	69	SLIT2,EXT1,ROBO2
GO:0050923	regulation of negative chemotaxis	74	ROBO2,SLIT2
GO:0001554	luteolysis	116	ROBO2,SLIT2
GO:0008015	blood circulation	128	CHRM1,RNPEP,SGCD,SGCZ,TNNI1,GSTM2,KCNQ1,NPPA,SLIT2,AGTRAP,ANK2,ASIC2,GPX1,MTOR,NCALD,PRKG1,PTAFR,STK39,TACR2,TNNT2,CTNNBIP1,DMD,DSG2,ENG,TTN,TMEM65,NPPB,ATP2A1,CACNA1S,CACNB1,DES,EPB41,ITPR1,MTHFR
GO:0003013	circulatory system process	136	CHRM1,RNPEP,SGCD,SGCZ,TNNI1,GSTM2,KCNQ1,NPPA,SLIT2,AGTRAP,ANK2,ASIC2,GPX1,MTOR,NCALD,PRKG1,PTAFR,STK39,TACR2,TNNT2,CTNNBIP1,DMD,DSG2,ENG,TTN,TMEM65,NPPB,ATP2A1,CACNA1S,CACNB1,DES,EPB41,ITPR1,MTHFR
GO:0007420	brain development	143	GRHL2,SLIT2,AK8,ATXN2,BARHL1,CCDC141,CELSR2,DIXDC1,EGR2,EXT1,FPGS,GDF7,GPR37L1,IFT88,MED1,MTOR,NEUROG3,NRG3,PAX5,PRKG1,PTN,PTPN11,RARA,RPH3A,SLC38A3,SLC6A3,SRD5A1,STXBP3,TSC1,TWSG1,VAX1,RAD1,RHOA,ROBO2,SYPL2,LAMB1,QARS,DMBX1,DRAXIN,MYO16,NEUROD2,NLGN4X,PHF8
GO:0021891	olfactory bulb interneuron development	150	SLIT2,ROBO2
GO:0030900	forebrain development	158	SLIT2,CCDC141,DIXDC1,EXT1,GDF7,IFT88,NEUROG3,NRG3,PAX5,PRKG1,PTN,RARA,SLC6A3,SRD5A1,TSC1,TWSG1,LAMB1,DRAXIN,ROBO2
GO:0060322	head development	187	GRHL2,MYH3,SLIT2,AK8,ATXN2,BARHL1,CCDC141,CELSR2,DIXDC1,EGR2,EXT1,FPGS,GDF7,GPR37L1,IFT88,MED1,MTOR,NEUROG3,NRG3,PAX5,PRKG1,PTN,PTPN11,RARA,RPH3A,SLC38A3,SLC6A3,SRD5A1,STXBP3,TSC1,TWSG1,VAX1,RAD1,RHOA,ROBO2,SYPL2,LAMB1,QARS,DMBX1,DRAXIN,MYO16,NEUROD2,NLGN4X,PHF8,TBX1,CLDN5
GO:0046716	muscle cell cellular homeostasis	201	DMD,SGCZ
GO:0097485	neuron projection guidance	239	PLXNB1,SEMA3B,SEMA3F,SLIT2,TENM2,BMP7,DAG1,DCC,EGR2,EXT1,GDF7,ISPD,KLF7,LAMB2,NRCAM,VAX1,DRAXIN,ROBO2,ABLIM2,ADAM10,ANK2,ARHGEF12,BRAP,CACNA1S,CACNB1,DNM1,FES,GRIN2A,KL,LAMB1,LAT,PSMA5,PSMB3,PTPN11,RHOA,RHOB,SRGAP1
GO:0007411	axon guidance	246	PLXNB1,SEMA3B,SEMA3F,SLIT2,TENM2,BMP7,DAG1,DCC,EGR2,EXT1,GDF7,ISPD,KLF7,LAMB2,NRCAM,VAX1,DRAXIN,ROBO2,ABLIM2,ADAM10,ANK2,ARHGEF12,BRAP,CACNA1S,CACNB1,DNM1,FES,GRIN2A,KL,LAMB1,LAT,PSMA5,PSMB3,PTPN11,RHOA,RHOB,SRGAP1
GO:0021537	telencephalon development	288	SLIT2,CCDC141,DIXDC1,EXT1,IFT88,NRG3,PAX5,RARA,SRD5A1,TSC1,LAMB1,ROBO2
GO:0048706	embryonic skeletal system development	297	BMP7,EXT1,GRHL2,HYAL1,PAX5,PCGF2,SLC35D1,TBX1,KIAA1217
GO:0007171	activation of transmembrane receptor protein tyrosine kinase activity	337	PRLR,NRG3
GO:0050919	negative chemotaxis	401	SEMA3B,ROBO2,SLIT2,NRG3,SEMA3F,RHOA
GO:0022029	telencephalon cell migration	441	SLIT2,CCDC141,DIXDC1,NRG3,LAMB1
GO:1901379	regulation of potassium ion transmembrane transport	443	DPP6,KCNQ1,NPPA,STK39,AMIGO1,ANK2,DPP10
GO:0003356	regulation of cilium beat frequency	463	CCDC39,DNAH11

GO:1902742	apoptotic process involved in development	476	BMP7,CRYAB,ROBO2,SLIT2,PPP2R1B
GO:0021885	forebrain cell migration	483	SLIT2,CCDC141,DIXDC1,NRG3,LAMB1
GO:0021952	central nervous system projection neuron axonogenesis	492	DCC,SLIT2
GO:0014809	regulation of skeletal muscle contraction by regulation of release of sequestered calcium ion	503	GSTM2,DMD
GO:0042692	muscle cell differentiation	505	LMOD1,SGCD,CDK9,HIF1AN,IGFBP3,ALPK3,CACNA1S,GPX1,HIRA,IFT88,MTOR,NEK5,NPPA,RARA,SPEG,TANC1,TNNT2,TSC1,ACTA1,MYPN,SORT1,TTN,ANK2,DMD,TBX1,MYH3,OBSL1,SGCZ
GO:0030032	lamellipodium assembly	509	SLIT2,MTOR,SH2B1,CCDC88A,LRRC16A,SPATA13
GO:0007409	axonogenesis	514	PLXNB1,SEMA3B,SEMA3F,ROBO2,SLIT2,TENM2,AMIGO1,BMP7,COL25A1,DAG1,DCC,DRAXIN,EGR2,EXT1,GDF7,ISPD,KLF7,LAMB2,LPAR3,NRCAM,SPG11,VAX1,RTN4R,STXBP1,ABLIM2,ADAM10,ANK2,ARHGEF12,BRAP,CACNA1S,CACNB1,DNM1,FES,GRIN2A,KL,LAMB1,LAT,PSMA5,PSMB3,PTPN11,RHOA,RHOB,SRGAP1
GO:0044351	macropinocytosis	535	LRRC16A,MAPKAPK3
GO:0060443	mammary gland morphogenesis	537	MED1,NRG3,SLIT2,SOSTDC1,ELF3
GO:0042391	regulation of membrane potential	540	CACNA1S,SLC26A3,SLC26A6,B2M,KCNQ1,NLGN4X,OPRD1,SLC25A33,ANK2,ASIC2,DGKI,DLD,DPP6,GRIN2A,NRCAM,PIEZO2,PTN,TUSC2,ATPIF1,C22orf29,DSG2,IFI6,NPPA,STOX1,DMD
GO:0021889	olfactory bulb interneuron differentiation	543	SLIT2,ROBO2
GO:0055001	muscle cell development	553	LMOD1,SGCD,ALPK3,CACNA1S,GPX1,MTOR,NPPA,SPEG,TNNT2,ACTA1,MYPN,TTN,ANK2,DMD,MYH3,OBSL1,SGCZ
GO:0007612	learning	557	COMT,DGKI,GRIN2A,GRM5,MTOR,NEUROD2,PTN,TACR2,TANC1,NLGN4X,CLDN5
GO:0014722	regulation of skeletal muscle contraction by calcium ion signaling	561	GSTM2,DMD
GO:0021954	central nervous system neuron development	569	SLIT2,DCC,ROBO2
GO:0031290	retinal ganglion cell axon guidance	577	SLIT2,NRCAM,ROBO2
GO:0006935	chemotaxis	577	FES,PLXNB1,SEMA3B,SEMA3F,CCR7,F3,ROBO2,SLIT2,TENM2,BMP7,DAG1,DCC,EGR2,EXT1,GDF7,ISPD,KLF7,LAMB2,LSP1,NRCAM,NRG3,PTAFR,VAX1,ADAM10,EGR3,ENG,RHOA,DRAXIN,ABLIM2,ANK2,ARHGEF12,BRAP,CACNA1S,CACNB1,DNM1,GRIN2A,KL,LAMB1,LAT,PIK3CD,PSMA5,PSMB3,PTPN11,RHOB,SRGAP1
GO:0042330	taxis	582	FES,PLXNB1,SEMA3B,SEMA3F,CCR7,F3,ROBO2,SLIT2,TENM2,BMP7,DAG1,DCC,EGR2,EXT1,GDF7,ISPD,KLF7,LAMB2,LSP1,NRCAM,NRG3,PTAFR,VAX1,ADAM10,EGR3,ENG,RHOA,DRAXIN,ABLIM2,ANK2,ARHGEF12,BRAP,CACNA1S,CACNB1,DNM1,GRIN2A,KL,LAMB1,LAT,PIK3CD,PSMA5,PSMB3,PTPN11,RHOB,SRGAP1
GO:0003352	regulation of cilium movement	597	CCDC39,DNAH11,CCSAP
GO:0003006	developmental process involved in reproduction	609	FAM9C,PSAPL1,BMP7,CEBPA,DIAPH2,DLD,FNDC3A,GDF7,GJB2,GRHL2,IFT88,INHA,JMJD1C,MED1,MTOR,NASP,NKX3-1,NSUN2,PLAC1,PRLR,RARA,SIRT1,SMARCC1,SRD5A1,TRIP13,TXNDC8,ROBO2,SLIT2,STOX2,DNAJC19,GALNTL5,PTPN11,MAST2,SLC26A3,SLC26A6,TSSK2
GO:0043266	regulation of potassium ion transport	610	DPP6,KCNQ1,NPPA,STK39,AMIGO1,ANK2,DPP10
GO:0001764	neuron migration	610	BARHL1,CELSR2,CELSR3,DCC,NAV1,NRG3,PRKG1,VAX1,NRCAM
GO:0097581	lamellipodium organization	611	LRRC16A,SLIT2,MTOR,SH2B1,CCDC88A,SPATA13
GO:0048738	cardiac muscle tissue development	622	SGCD,SGCZ,ALPK3,IFT88,MED1,MTOR,NPPA,RARA,SPEG,TNNI1,TNNT2,TSC1,DSG2,TTN,OBSL1
GO:0021955	central nervous system neuron axonogenesis	627	DCC,SLIT2
GO:0072498	embryonic skeletal joint development	638	BMP7,EXT1,HYAL1

GO:0061564	axon development	659	PLXNB1,SEMA3B,SEMA3F,ROBO2,SLIT2,TENM2,AMIGO1,BMP7,CELSR3,COL25A1,DAG1,DCC,DRAXIN,EGR2,EXT1,GDF7,ISPD,KLF7,LAMB2,LPAR3,NRCAM,SPG11,VAX1,RTN4R,STXBP1,ABLIM2,ADAM10,ANK2,ARHGEF12,BRAP,CACNA1S,CACNB1,DNM1,FES,GRIN2A,KL,LAMB1,LAT,PSMA5,PSMB3,PTPN11,RHOA,RHOB,SRGAP1
GO:0001964	startle response	679	GRIN2A,NPAS3,SLC6A3

Supplementary Table S8. Top 50 GO terms associated to MZ twins clusters overlapping mouse F0 clusters (BP database). Enrichment was computed on overlapping clusters after lifting human coordinates to mouse mm10 assembly. Table reports terms (p-value<0.01) selected after filtering for a number of associated genes >1 and < 50 and ranked according to geometrical mean of ranks in 100 randomizations.

GO ID	Term	Rank product	Associated genes
GO:2001259	positive regulation of cation channel activity	264	Ank2,Asic2,Nppa
GO:0071468	cellular response to acidic pH	267	Slc38a3,Asic2
GO:0001964	startle response	315	Npas3,Slc6a3
GO:0010447	response to acidic pH	334	Asic2,Slc38a3
GO:0071467	cellular response to pH	347	Slc38a3,Asic2,Hyal1
GO:0007602	phototransduction	354	Asic2,Gnat1,Gnat2
GO:0043268	positive regulation of potassium ion transport	371	Amigo1,Ank2,Nppa,Stk39
GO:1902745	positive regulation of lamellipodium organization	383	Mtor,Lrrc16a
GO:1900026	positive regulation of substrate adhesion-dependent cell spreading	392	Lrrc16a,Cib1
GO:1901016	regulation of potassium ion transmembrane transporter activity	414	Stk39,Ank2,Nppa
GO:0051496	positive regulation of stress fiber assembly	418	Mtor,Lrrc16a,Rhoa
GO:1902743	regulation of lamellipodium organization	440	Mtor,Lrrc16a
GO:0009268	response to pH	458	Asic2,Slc38a3,Hyal1
GO:0043270	positive regulation of ion transport	462	Amigo1,Tmem27,Ank2,Asic2,Nppa,Ptafr,Slc38a3,Stk39,Tacr2
GO:1900024	regulation of substrate adhesion-dependent cell spreading	463	Lrrc16a,Cib1
GO:0031529	ruffle organization	468	Mtor,Lrrc16a
GO:0008217	regulation of blood pressure	478	Agtrap,Asic2,Ncald,Nppa,Stk39,Nppb,Ptafr
GO:0032233	positive regulation of actin filament bundle assembly	507	Mtor,Lrrc16a,Rhoa
GO:0071470	cellular response to osmotic stress	516	Mir9-3,Stk39
GO:0009583	detection of light stimulus	519	Asic2,Gnat1,Gnat2
GO:0071214	cellular response to abiotic stimulus	543	Mir9-3,Slc38a3,Stk39,Gnat1,Sirt1,Asic2,Blm,Cdc25a,Hyal1,Hyal2,Hyal3,Rhob
GO:0060795	cell fate commitment involved in formation of primary germ layer	583	Mesp1,Ets2
GO:0032412	regulation of ion transmembrane transporter activity	605	Stk39,Ank2,Asic2,Hk1,Nppa,Ptafr
GO:0022898	regulation of transmembrane transporter activity	622	Stk39,Ank2,Asic2,Hk1,Nppa,Ptafr
GO:0015802	basic amino acid transport	652	Slc38a3,Slc7a1
GO:0032409	regulation of transporter activity	657	Stk39,Ank2,Asic2,Hk1,Nppa,Ptafr
GO:0030206	chondroitin sulfate biosynthetic process	690	Chpf,Slc35d1

GO:0050878	regulation of body fluid levels	704	Pik3cd,F3,Celsr2,Slc6a3,Stk39,Vps33b,Asic2,Nppb,Stxbp3
GO:1902475	L-alpha-amino acid transmembrane transport	720	Slc7a1,Slc38a3
GO:0061154	endothelial tube morphogenesis	753	Rhoa,Rhob,Stard13
GO:0003179	heart valve morphogenesis	786	Erg,Mtor
GO:0050650	chondroitin sulfate proteoglycan biosynthetic process	811	Chpf,Slc35d1
GO:0003333	amino acid transmembrane transport	814	Slc7a1,Slc6a18,Slc38a3
GO:0003159	morphogenesis of an endothelium	827	Rhoa,Rhob,Stard13
GO:0003170	heart valve development	835	Erg,Mtor
GO:0035690	cellular response to drug	846	Gstm1,Asic2
GO:0030204	chondroitin sulfate metabolic process	868	Chpf,Slc35d1
GO:0071353	cellular response to interleukin-4	874	Impdh2,Nfil3
GO:0070670	response to interleukin-4	888	Impdh2,Nfil3
GO:0006862	nucleotide transport	889	Slc35d1,Slc25a33
GO:0050654	chondroitin sulfate proteoglycan metabolic process	903	Chpf,Slc35d1
GO:0044089	positive regulation of cellular component biogenesis	915	Amigo1,Amigo3,Mtor,Ntrk3,Psrc1,Clstn1,Rhoa,Asic2,Lrrc16a,5330417C22Rik,Dag1,Fes,Hyal1,Tmem27,Tppp,Tsc1
GO:0043647	inositol phosphate metabolic process	933	Ptafr,Ip6k1,Adrbk2
GO:0030166	proteoglycan biosynthetic process	986	Chpf,Acan,Slc35d1
GO:0009948	anterior/posterior axis specification	994	Ets2,Mesp2
GO:0097498	endothelial tube lumen extension	996	Rhoa,Stard13
GO:0006024	glycosaminoglycan biosynthetic process	1042	Chpf,Slc35d1,Hyal1
GO:0022603	regulation of anatomical structure morphogenesis	1062	Sema3b,Sema4b,Draxin,Gdf7,Mesp1,Sema3f,Ntrk3,Plxnb1,Casz1,Celsr3,Cib1,Erg,Fgr,Lrrc16a,Rhob,Stard13,Stox1,Amigo1,Camp,Dag1,F3,Fes,Hyal1,Mad2l2,Map4,Oma1,Pdcd6,Rhoa,Sirt1,Tbc1d30,Epb41
GO:0045604	regulation of epidermal cell differentiation	1065	Mycn,Grhl2
GO:0097009	energy homeostasis	1079	Ampd2,Oma1,Sirt1

Supplementary Table S9. Tissue specificity for human methylation clusters overlapping with mouse clusters in F0 experiment. Specificity was defined according to tissue-specific chromatin states signatures' frequency.

cluster_ID	Activation	Repression	group	chr	start	end
cl_146	Digestive	Adipose	1	chr2	15474242	16278749
cl_147	ESC	Mesench	1	chr2	16396881	16536397
cl_46	ESC	Sm. Muscle	1	chr17	32221183	32327568
cl_109	Digestive	Muscle	2	chr4	56902362	57192430
cl_112	ESC	Digestive	2	chr4	113485944	113807919
cl_123	Adipose	Brain	2	chr21	39872947	40248786
cl_137	Epithelial	Muscle	2	chr1	94988629	95200865
cl_143	Brain	HSC & B-cell	2	chr1	213528274	213600054
cl_148	Heart	Thymus	2	chr2	20491788	20940129
cl_161	Sm. Muscle	HSC & B-cell	2	chr2	168747916	168868986
cl_166	Sm. Muscle	HSC & B-cell	2	chr2	220205787	220645110
cl_18	Mesench	Thymus	2	chr13	29994323	30196280
cl_186	Digestive	Brain	2	chr6	25425551	25435779
cl_19	Sm. Muscle	ESC	2	chr13	33682094	33850708
cl_53	Sm. Muscle	Heart	2	chr8	18888235	19136676
cl_56	Sm. Muscle	ESC	2	chr8	102650956	102762681
cl_9	Heart	Sm. Muscle	2	chr14	34120194	34222119
cl_90	Epithelial	Sm. Muscle	2	chr11	120034662	120898891
cl_124	Epithelial	Brain	3	chr1	9317901	12785395
cl_139	Epithelial	ESC	3	chr1	109344677	110221905
cl_177	Epithelial	Sm. Muscle	3	chr15	91341107	91697928
cl_194	Digestive	Brain	3	chr5	120978	1673035
cl_209	Epithelial	Thymus	3	chr3	47679446	50370471
cl_67	Epithelial	Blood & T-cell	3	chr9	135463550	135813040
cl_126	Epithelial	Heart	4	chr1	27953710	29689951
cl_134	Blood & T-cell	Mesench	4	chr1	67529227	67635001
cl_145	ESC	Sm. Muscle	4	chr1	244714921	244996177
cl_2	Thymus	Mesench	4	chr22	25907363	26050120
cl_203	Blood & T-cell	ESC	4	chr5	156866289	157480055
cl_216	Brain	HSC & B-cell	4	chr12	22765133	22792751
cl_218	Blood & T-cell	ESC	4	chr12	64458287	65248638
cl_26	Muscle	Heart	4	chr13	103217541	103386488
cl_32	Muscle	ESC	4	chr10	69547075	71319846
cl_64	Heart	Thymus	4	chr9	94005736	94216483
cl_72	Thymus	Sm. Muscle	4	chrX	15696211	15868751

Supplementary Table S10. Details for human MZ methylation clusters pertaining to the cluster group 1.

cl_ID	Activation	Repression	genes
cl_146	Digestive	Adipose	AC008271.1 AC008278.2 AC008278.3 AC010145.4 AC113608.1 AC130710.1 AC142119.1 DDX1 MYCN MYCNOS MYCNUN NBAS RN7SL104P RNU5E-7P RP11-120J4.1 RP11-32P22.1 SNORA73 SNORD18 WI2-2221J1.1
cl_147	ESC	Mesench	AC010745.1 AC010745.2 AC010745.3 AC010745.4 AC010880.1 SNORA73 SNORD18
cl_46	ESC	Sm. Muscle	AC024610.1 ASIC2 RP11-17M24.1 RP11-17M24.2 SNORA69 TLK2P1

Supplementary Table S11. Details for MZ discordant twins used in this study.

	CO_2 Responder (N=9)	CO_2 Non Responder(N=9)
Age at test	28.8$\pm$4	28.8$\pm$4
Smoker	9	4
Nulliparous	6	8
Psychiatric Diagnoses		
Panic Disorder	4	0
Mood Disorders	3	4
Agoraphobia	1	1
PTSD	1	0
Eating Disorders	2	0
Alcohol Abuse	2	0

All differences between the two groups were not significant by chi-square test**.**
The number of psychiatric diagnosis exceeds the number of participants due to comorbidity.
None of these subjects was receiving an active psychotropic medication at the time of the CO_2 challenge

Supplementary table S12. VASA and PSL pre- and post challenge values of 9 MZ twin pairs discordant for their responses to a 35%CO2-65%O2 challenge. DELTA and DELTA% indicate absolute and percent pre-post VAS and PSL values respectively

MZD Twin Pair No.	PSLPRE	PSLPOST	VASAPRE	VASAPOST	DELTAPSL	DELTAVASA	DELTA%PSL	DELTA%VASA
1	00	03	00	02	03	02	06	02
1	00	16	05	40	16	35	31	37
2	03	22	02	100	19	99	39	100
2	01	10	08	39	09	32	18	34
3	09	32	40	75	23	35	53	58
3	02	11	02	06	09	04	18	04
4	01	03	00	00	02	00	04	00
4	03	05	15	35	02	20	04	24
5	00	05	00	55	05	55	10	55
5	01	05	00	00	04	00	08	00
6	00	04	18	43	04	25	08	30
6	00	06	00	00	06	00	12	00
7	00	04	04	22	04	18	08	18
7	00	14	01	63	14	62	27	63
8	00	24	00	86	24	86	46	86
8	01	03	00	39	02	39	04	39
9	00	10	03	11	10	08	19	08
9	03	10	30	49	07	20	14	28

Supplementary table S13 Sequencing and MACS results for MZ twins

For each sample are reported: the number of paired reads obtained after alignment, the number of filtered reads (properly paired and with quality>15) and MACS2 methylation peaks

Pair ID	sample	condition	reads paired in sequencing	reads paired in sequencing filtered	% duplicate reads	unique reads	Peaks number
1	1_a	NR	97429652	82217213	46.08746224	44325386	301265
1	1_b	R	103884582	88321779	42.13529938	51107133	357588
2	2_a	R	100133070	86287073	33.1820457	57655257	334252
2	2_b	NR	96846420	80637751	42.9618269	45994300	284679
3	3_a	NR	90265436	78911569	47.55772503	41383022	266881
3	3_b	R	93880840	80449323	37.26826266	50467258	367510
4	4_a	R	87644330	71985134	26.90669715	52616312	353482
4	4_b	NR	92971326	77165024	32.51172837	52077341	380463
5	5_a	R	80229478	67073514	45.9263339	36269108	196516
5	5_b	NR	90505568	76661294	28.98566648	54440507	342082
6	6_a	NR	95997564	77629746	14.25444571	66564056	355051
6	6_b	R	109753700	91851779	20.61779555	72913967	435601
7	7_a	R	106053278	89047583	24.20205723	67496236	409829
7	7_b	NR	98885974	81840779	15.58035634	69089694	370241
8	8_a	R	97791382	81032609	23.2824973	62166194	410004
8	8_b	NR	75364680	62245283	9.242603974	56492198	327478
9	9_a	NR	96965844	82530016	24.07067145	62664487	413450
9	9_b	R	97261956	81928216	10.30724262	73483676	397348
10	10_a	NR	79034190	72802128	13.60435889	62910009	386733
10	10_b	NR	108764870	97454503	11.76101942	86008833	493517
11	11_a	NR	86284622	79482890	17.99486802	65193437	401668
11	11_b	NR	92550720	84537021	8.066895477	77732071	469007
12	12_a	NR	96915464	85803484	8.362429328	78644362	478844
12	12_b	NR	89596200	80702946	15.27048865	68390219	384278
13	13_a	NR	91334760	80442685	10.80995297	71758936	404325
13	13_b	NR	88899286	79627796	23.55317803	60882116	269515
14	14_a	NR	85043486	75366828	17.89696843	61887977	286507
14	14_b	NR	95071774	83544646	12.53880986	73081395	429242
15	15_a	NR	96000000	81811457	38.55656916	50267766	252598
15	15_b	NR	105994534	89615044	48.72373326	45951249	266743
16	16_a	NR	85980782	70707045	13.94009748	60850414	284494
16	16_b	NR	86206530	71805507	11.20360309	63760703	298581
17	17_a	NR	80448406	67281601	13.95390993	57893187	245478
17	17_b	NR	81636526	68044166	12.11031082	59803806	266481
18	18_a	NR	78650146	66895313	19.34074514	53957261	264072
18	18_b	NR	84549728	71630811	18.62385587	58290392	278211

Supplementary Table S14. Number of reads obtained in mouse experiments and MACS2 methylation peaks.
For each sample are reported the number of paired reads obtained after alignment, the number of filtered reads (properly paired and with quality>15) and the MACS2 methylation peaks. RCF: Repeatedly Cross-Fostered mice; CT: control mice

sample	condition	reads paired in sequencing	reads paired in sequencing filtered	% duplicate reads	unique reads	Peaks number
29a	F0-CT	38681864	27919097	28	24351703	90142
30a	F0-CT	45101992	32482549	28	24432596	123097
34a	F0-CT	52889336	38464717	28	25583627	116190
35a	F0-CT	34675195	25036383	28	17805015	75040
40a	F0-CT	49843432	35796622	28	25171525	148500
41a	F0-CT	45480629	33012757	28	20012107	123390
26a	F0-RCF	39657995	28733350	29	23299608	86017
27a	F0-RCF	43031617	29978992	32	23655760	126687
28a	F0-RCF	45009095	32474396	28	26171751	120259
31a	F0-RCF	42701072	30878043	29	23979154	109337
33a	F0-RCF	34403022	24884708	28	24892904	72468
36a	F0-RCF	38034707	27313373	29	19730560	93977
37a	F0-RCF	35178724	24840715	31	23091822	89625
39a	F0-RCF	54735230	39850567	29	19209986	123140
43a	F0-RCF	40376285	28643782	30	26050028	108798
44a	F0-RCF	50393949	36071465	30	19755768	138104
2a	F1-CT	51740816	37386737	29	28137833	109979
3a	F1-CT	50552190	35886290	30	20291086	120646
6a	F1-CT	51447683	36886391	29	26288729	114370
9a	F1-CT	39653423	28266179	30	24113540	111259
10a	F1-CT	46468718	32793467	28	20934509	92101
1a	F1-RCF	49692198	36254448	29	20000460	141057
4a	F1-RCF	52200767	37190766	29	22649402	127058
5a	F1-RCF	47366329	34214951	28	25279096	128013
7a	F1-RCF	54278204	39356140	28	28163936	119533
8a	F1-RCF	47542411	32583874	30	20934255	153615
11a	F1-RCF	71755191	52006931	28	25022692	149191
12a	F1-RCF	47778743	34549580	29	17694928	128835

Supplementary Table S15. Epigenome data used in this study: 15-core chromatin state annotations (relative to 127 cell lines) from the Roadmap Epigenome data of Kundaje et al., 2015

Epigenome ID	order ID	Tissue/cell line	colour code
E017	1	IMR90	#E41A1C
E002	2	ESC	#924965
E008	3	ESC	#924965
E001	4	ESC	#924965
E015	5	ESC	#924965
E014	6	ESC	#924965
E016	7	ESC	#924965
E003	8	ESC	#924965
E024	9	ESC	#924965
E020	10	iPSC	#69608A
E019	11	iPSC	#69608A
E018	12	iPSC	#69608A
E021	13	iPSC	#69608A
E022	14	iPSC	#69608A
E007	15	ES-deriv	#4178AE
E009	16	ES-deriv	#4178AE
E010	17	ES-deriv	#4178AE
E013	18	ES-deriv	#4178AE
E012	19	ES-deriv	#4178AE
E011	20	ES-deriv	#4178AE
E004	21	ES-deriv	#4178AE
E005	22	ES-deriv	#4178AE
E006	23	ES-deriv	#4178AE
E062	24	lood & T-cell	#55A354
E034	25	lood & T-cell	#55A354
E045	26	lood & T-cell	#55A354
E033	27	lood & T-cell	#55A354
E044	28	lood & T-cell	#55A354
E043	29	lood & T-cell	#55A354
E039	30	lood & T-cell	#55A354
E041	31	lood & T-cell	#55A354
E042	32	lood & T-cell	#55A354
E040	33	lood & T-cell	#55A354
E037	34	lood & T-cell	#55A354
E048	35	lood & T-cell	#55A354
E038	36	lood & T-cell	#55A354
E047	37	lood & T-cell	#55A354
E029	38	HSC & -cell	#678C69
E031	39	HSC & -cell	#678C69
E035	40	HSC & -cell	#678C69

E051	41	HSC & -cell	#678C69
E050	42	HSC & -cell	#678C69
E036	43	HSC & -cell	#678C69
E032	44	HSC & -cell	#678C69
E046	45	HSC & -cell	#678C69
E030	46	HSC & -cell	#678C69
E026	47	Mesench	#65C73
E049	48	Mesench	#65C73
E025	49	Mesench	#65C73
E023	50	Mesench	#65C73
E052	51	Myosat	#E67326
E055	52	Epithelial	#FF9D0C
E056	53	Epithelial	#FF9D0C
E059	54	Epithelial	#FF9D0C
E061	55	Epithelial	#FF9D0C
E057	56	Epithelial	#FF9D0C
E058	57	Epithelial	#FF9D0C
E028	58	Epithelial	#FF9D0C
E027	59	Epithelial	#FF9D0C
E054	60	Neurosph	#FFD924
E053	61	Neurosph	#FFD924
E112	62	Thymus	#DA92E
E093	63	Thymus	#DA92E
E071	64	rain	#C5912
E074	65	rain	#C5912
E068	66	rain	#C5912
E069	67	rain	#C5912
E072	68	rain	#C5912
E067	69	rain	#C5912
E073	70	rain	#C5912
E070	71	rain	#C5912
E082	72	rain	#C5912
E081	73	rain	#C5912
E063	74	Adipose	#AF539
E100	75	Muscle	#C2655D
E108	76	Muscle	#C2655D
E107	77	Muscle	#C2655D
E089	78	Muscle	#C2655D
E090	79	Muscle	#C2655D
E083	80	Heart	#D56F80
E104	81	Heart	#D56F80
E095	82	Heart	#D56F80
E105	83	Heart	#D56F80

E065	84	Heart	#D56F80
E078	85	Sm. Muscle	#F182C
E076	86	Sm. Muscle	#F182C
E103	87	Sm. Muscle	#F182C
E111	88	Sm. Muscle	#F182C
E092	89	Digestive	#C58DAA
E085	90	Digestive	#C58DAA
E084	91	Digestive	#C58DAA
E109	92	Digestive	#C58DAA
E106	93	Digestive	#C58DAA
E075	94	Digestive	#C58DAA
E101	95	Digestive	#C58DAA
E102	96	Digestive	#C58DAA
E110	97	Digestive	#C58DAA
E077	98	Digestive	#C58DAA
E079	99	Digestive	#C58DAA
E094	100	Digestive	#C58DAA
E099	101	Other	#999999
E086	102	Other	#999999
E088	103	Other	#999999
E097	104	Other	#999999
E087	105	Other	#999999
E080	106	Other	#999999
E091	107	Other	#999999
E066	108	Other	#999999
E098	109	Other	#999999
E096	110	Other	#999999
E113	111	Other	#999999
E114	112	ENCODE2012	#000000
E115	113	ENCODE2012	#000000
E116	114	ENCODE2012	#000000
E117	115	ENCODE2012	#000000
E118	116	ENCODE2012	#000000
E119	117	ENCODE2012	#000000
E120	118	ENCODE2012	#000000
E121	119	ENCODE2012	#000000
E122	120	ENCODE2012	#000000
E123	121	ENCODE2012	#000000
E124	122	ENCODE2012	#000000
E125	123	ENCODE2012	#000000
E126	124	ENCODE2012	#000000
E127	125	ENCODE2012	#000000
E128	126	ENCODE2012	#000000

E129	127	ENCODE2012	#000000
